# Single-cell RNA-seq variant analysis for exploration of genetic heterogeneity in cancer

**DOI:** 10.1038/s41598-019-45934-1

**Published:** 2019-07-02

**Authors:** Erik Fasterius, Mathias Uhlén, Cristina Al-Khalili Szigyarto

**Affiliations:** 1 School of Chemistry, Biotechnology and Health, KTH Royal Institute of Technology, Stockholm, Sweden; 2 Science for Life Laboratory, KTH Royal Institute of Technology, Solna, Sweden

**Keywords:** Cancer genomics, Next-generation sequencing

## Abstract

Inter- and intra-tumour heterogeneity is caused by genetic and non-genetic factors, leading to severe clinical implications. High-throughput sequencing technologies provide unprecedented tools to analyse DNA and RNA in single cells and explore both genetic heterogeneity and phenotypic variation between cells in tissues and tumours. Simultaneous analysis of both DNA and RNA in the same cell is, however, still in its infancy. We have thus developed a method to extract and analyse information regarding genetic heterogeneity that affects cellular biology from single-cell RNA-seq data. The method enables both comparisons and clustering of cells based on genetic variation in single nucleotide variants, revealing cellular subpopulations corroborated by gene expression-based methods. Furthermore, the results show that lymph node metastases have lower levels of genetic heterogeneity compared to their original tumours with respect to variants affecting protein function. The analysis also revealed three previously unknown variants common across cancer cells in glioblastoma patients. These results demonstrate the power and versatility of scRNA-seq variant analysis and highlight it as a useful complement to already existing methods, enabling simultaneous investigations of both gene expression and genetic variation.

## Introduction

Cancer is among the leading causes of disease-related deaths worldwide, but increasing knowledge regarding tumour heterogeneity and molecular alterations can facilitate the development of novel, personalised treatments^1^. Cancer is an evolutionary process where mutations arise and accumulate in normal cells, leading to a selective growth advantage and tumour formation^2^. The biological changes that occur in a cell during cancer transformation have been studied extensively, which includes *e*.*g*. unresponsiveness to extracellular signals, uncontrolled proliferation, reduction and evasion of tumour suppression, formation of blood vessels and, in the later stages, development of metastases^3^. Understanding tumour heterogeneity and the mutations leading to functional aberrations of expressed genes is thus a critical goal of cancer research and development of personalised medicine. The KRAS oncogene is an example of how important knowledge of specific mutations is, as it possesses several well-known variants that are known to prevent anti-EGFR treatments from being effective^4^.

Genetic heterogeneity between tumours is one of the largest problems for finding a effective cancer treatments, as no single drug is likely to work for every cancer type^5^. This is not limited to inter-patient variation, as a single tumour gives rise to sub-populations of cells that acquire different mutations both over time and in space^2^. There is an ongoing debate as to how this intra-tumour heterogeneity is formed: different models of tumour progression have been identified, such as linear (where acquired driver mutations in single cells confer a selective advantage) or branching models (where different sub-clones diverge from a common ancestor and then evolve in parallel)^2,6^. Cancerous cells accumulate mutations that alter biological functions, leading to the development of tumour phenotypes that can circumvent the effect of therapy and cause relapse^7,8^. Genetic heterogeneity at the DNA-level has consequences for both transcripts and proteins, affecting the expression and function of RNA and proteins. While any mutation can potentially affect gene expression through *e*.*g*. promoter regions, mutations that are transcribed to RNA likely affect the functionality of their final protein products. Mutations residing in introns influence splicing events and exon composition of translated isoforms, whereas mutations in exons may ultimately influence both protein sequence and function through changes in *e*.*g*. structure, level of activity or ability to interact with other proteins.

RNA sequencing (RNA-seq) has been used to heterogeneity and gene expression variation across a multitude of samples, conditions and diseases^9–11^. The RNA-seq technology is robust, yields data with single base pair resolution, low background signals and a has wide dynamic detection range^12–14^. Advanced systems for single cell RNA-seq (scRNA-seq) have used individual expression profiles of single cells to reveal inter- and intra-tumour heterogeneity and cellular sub-populations within tumours^15–18^.

RNA-seq analyses usually focus on gene expression exclusively, which leaves the sequence information unused. It is, however, becoming more common to utilise this information^19–22^. The usefulness of RNA-seq variant analysis is demonstrated by its application on a wide range of biological questions in numerous studies, including investigations of specific variants^19,20,23^, allele-specific expression^21^, and detection of micro-fluidic droplets containing more than one cell for single-cell sorting^24^. The sequence information gained from RNA-seq experiments can thus provide data on *e*.*g*. single nucleotide variants (SNVs), insertions and deletions in the same manner as *e*.*g*. exome sequencing, allowing for investigations into genetic heterogeneity expressed at the RNA-level. Such analyses could yield information regarding not only the expression-level heterogeneity already given by traditional RNA-seq analyses, but also how sequence variation affects protein functionality.

We have previously demonstrated how RNA-seq SNV analyses can be used for evaluating both cell line authenticity^25^ and genetic heterogeneity in public cell line^26^ and organoid^27^ datasets. Variant analyses have also been performed on scRNA-seq data, which focused on the presence or absence of SNVs rather than their genotypes (*i*.*e*. the sequences of each individual alleles for a given genomic position). Our methodology differs from others in that focuses on the genotype of each individual variant across all samples, yielding a measure of genetic heterogeneity without directly including gene expression itself. While the method has shown to be highly useful for bulk samples, it has yet to be evaluated on single-cell data.

In this study we demonstrate how genetic heterogeneity expressed on RNA-level vary both between and within patients by applying a genotype-centric SNV analysis method to two publicly available scRNA-seq datasets^17,18^. We highlight how such an analysis can quantify and characterise genetic heterogeneity expressed at the RNA-level, including how single-cell clustering based on this heterogeneity may be used to interrogate functionally distinct sub-populations within tumours. The single-cell variant analyses also highlight a discrepancy between genetic heterogeneity in core tumours and their metastastic derivatives, as well as high levels of variation in driver genes. Our methodology represents an extension of the already sizeable capacities of scRNA-seq, enabling researchers worldwide to utilise the full extent of the data that is generated by their experiments. Genotype-centric analyses of expressed genetic variation through scRNA-seq thus represents a useful complement to already existing methods.

## Results

### Analysing genetic heterogeneity using scRNA-seq data

Gene expression heterogeneity has been addressed in the context of many disorders and investigated using both bulk and single-cell RNA-seq data. While varying levels of gene expression across conditions or disease states are of vital importance, they do not capture the entire biological context within which they exist, *i*.*e*. when a mutation has impacted protein structure, stability or function. The sequence information from RNA-seq data could, however, also be utilised to investigate such genetic heterogeneity at the RNA-level. The present work aims to investigate how a genotype-centric scRNA-seq variant analysis can be used to explore tumour heterogeneity through transcribed SNVs and their impact on protein functionality. This is accomplished using two publicly available datasets covering different experimental designs, sequencing parameters and disease origins.

The first dataset is from a cohort of 11 breast cancer patients with approximately 50 cells per patient (henceforth referred to as the BC dataset)^17^, while the second covers four glioblastoma patients with 830 cells per patient (the GBM dataset)^18^; see Table S1. In addition to differing in number of patients and cells, the average sequencing depth per cell is approximately four times higher in the BC dataset. The BC dataset also contains metadata on cancer subtypes as well as two patients with lymph node metastases, while the GBM dataset contains gene expression-based cell type classifications. These two datasets thus cover a wide range of possible analyses and biological contexts, including both inter- and intra-patient heterogeneity as well as comparisons between core tumours *vis-à-vis* their metastatic derivatives, in addition to cell type clustering.

The raw data from both studies was aligned using the STAR 2-pass method^28^, followed by variant calling with the GATK RNA-seq Best Practices^29^ and lastly, annotation with snpEff^30^. The resulting variant calls were filtered based on several quality metrics (see the *Methods* section), resulting in medians of 9000 and 2000 SNVs per cell for the BC and GBM datasets, respectively. Pairwise sequence similarities were estimated using the previously established *similarity score*^26^, a weighted measure that incorporates both the number of matching genotypes between two cells as well as the total number of overlapping variants (*i*.*e*. positions with a confident variant call in both cells). Comparisons of SNVs were also performed between tumours and lymph node metastases where available (BC03 and BC07).

As can be seen in Tables 1 and S2, there is a wide range of global inter-patient genetic heterogeneity in both datasets but a larger variation for the GBM dataset, which spans similarity scores between 0.471 and 0.828 (compared to 0.615 to 0.750 for the BC dataset). Comparisons between heterogeneity in core tumours compared to metastases are also important, since they can provide information related to how aggressive tumours evolve. The differences between the tumours and the corresponding metastases of the BC dataset are both statistically significant ($P < 0.01$), showing that the metastases are less heterogeneous overall compared to their respective origins. This difference is the most pronounced for the BC07 patient, whose median similarity score is 0.714 and 0.750 for tumour and the metastasis, respectively, whereas the BC03 patient has a more modest difference (0.700 and 0.714). The inter-patient genetic heterogeneity for the GBM dataset is the highest for patient BT_S1 with a median similarity score of 0.471, while BT_S2 is the most stable at 0.828 (BT_S4 and BT_S6 has 0.621 and 0.636, respectively).

**Table 1:** Genetic heterogeneity in the BC dataset, with separate values for the lymph node metastases (“LN”) for the BC03 and BC07 patients.

Patient	Median similarity score	Cells	HIGH	MODERATE	LOW	MODIFIER
BC01	0.615	22	0.300	0.836	0.881	0.881
BC02	0.704	53	0.286	0.821	0.887	0.908
BC03	0.700	33	0.375	0.792	0.861	0.877
BC03LN	0.714*	53	0.444**	0.806*	0.853**	0.866**
BC04	0.698	55	0.375	0.804	0.884	0.89
BC05	0.682	75	0.500	0.845	0.884	0.891
BC06	0.750	18	0.400	0.812	0.867	0.884
BC07	0.714	50	0.286	0.797	0.872	0.892
BC07LN	0.750*	52	0.375**	0.809**	0.875*	0.893
BC08	0.688	22	0.167	0.760	0.852	0.923
BC09	0.643	55	0.375	0.779	0.749	0.899
BC10	0.643	15	0.286	0.812	0.796	0.921
BC11	0.682	11	0.400	0.849	0.890	0.902

*$P < 0.01$ (core tumour vs. lymph node).

**$P\, \lll \,0.01$ (core tumour vs. lymph node).

In order to further explore the characteristics of this genetic heterogeneity between and within patients, we sought to investigate whether separate variant impact categories contribute differently to the overall heterogeneity. Median similarity scores for each BC patient and location were calculated as previously, but subsetting for each individual variant impact category (Tables 1 and S3). There is a general increase in heterogeneity for higher impact variants, especially pronounced for the HIGH impact category. The higher level of heterogeneity in core tumours compared to metastases is conserved across all impacts for the BC07 patient (although this difference is not statistically significant for the MODIFIER category), while it is reversed for the LOW and MODIFIER categories for the BC03 patient. This indicates that the metastatic lesions show higher levels of conservation of variants likely affecting protein function compared to their respective core tumour.

There are additionally four breast cancer subtypes represented in the BC dataset: ER+, HER2+, ER+/HER2+ and triple negative breast cancer (TNBC). Information regarding genetic heterogeneity in different subtypes may contribute to knowledge about disease states in general and how their respective gene expression patterns relate to their mutational landscape. While a cohort of 11 patients is relatively small, it is nevertheless interesting to note that there is a pattern across the subtypes: the HER2+ and TNBC subtypes are generally more heterogeneous than ER+ and ER+/HER2+ (Table S4). The differences are statistically significant between all subtypes except ER+ vs. ER+/HER2+ and HER2+ vs. ER+/HER2+ (as estimated using ANOVA and Tukey’s HSD testing).

These results indicate that variant analysis on scRNA-seq data is both feasible and can yield biologically relevant information regarding genetic heterogeneity in core tumours and their metastatic derivatives as well as between different cancer subtypes.

### Using genetic heterogeneity for single-cell clustering

We also wanted to evaluate if a genotype-centric methodology could be used for clustering of single-cell data, which could be used to further evaluate different types of biological heterogeneity. Such clustering analyses are usually performed using RNA-seq expression data, but some studies have also used variants from both DNA and RNA data^31,32^. There is some variation in how such studies have chosen to perform the clustering, *e*.*g*. if non-overlapping variants are included or if gene expression measurements are incorporated. The previously published methods do not account for varying genotypes across samples, in lieu of focusing on presence or absence of variants overall. While such methodologies are easily applicable to a wide range of clustering strategies, they may inadvertently incorporate an error of unknown severity for samples with different genotypes for the same variant.

In order to test the potential of the genotype-centric methodology we first applied it on a previously described cohort of public bulk RNA-seq cell line datasets^26^. These were hierarchically clustered and evaluated used the *Adjusted Rand Index* (ARI, a measure of cluster performance), yielding a perfect index of 1 (Fig. S1). Using the similarity score for clustering thus shows high distinguishing power for bulk RNA-seq data.

An initial clustering of the single cell data yielded ARIs of 0.51 and 0.86 for the BC and GBM datasets, respectively (Fig. S2). This indicates that clustering of variant-based single-cell data may have higher performance for datasets with more cells per patient, rather than those that possess higher sequencing depth. These clusterings could also be optimised: ARIs upwards of 0.95 were found for the BC dataset by removing the MODIFIER impact category (*i*.*e*. variants predicted to have little to no effect on protein function, such as intergenic variants) and subsetting for variants not found in dbSNP (Figs S3 and S7).

**Figure 1:**
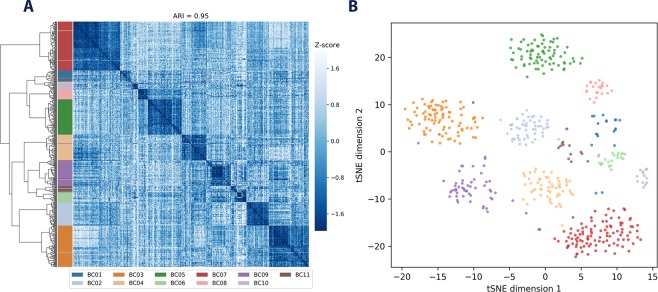
Cluster analysis of the BC dataset. A representative high-performing (**A**) hierarchical clustering and (**B**) tSNE of the BC dataset, where the MODIFIER impact category and variants contained in dbSNP have been excluded, as well as samples containing fewer than 50 variants in total.

In order to ascertain if other string-based distance metrics could also be used for single-cell variant clustering, the same evaluation scheme was also used for the concordance, Hamming and Levenshtein distances. The highest ARI for any of these metrics across either dataset was 0.67 (Figs S4–6 and S8–10), demonstrating that the similarity score performs well for variant-based clustering of single-cell data. A representative output of these higher-ARI clustering subsets using the similarity score is visualised in Fig. 1, clearly separating cells from the different patients into individual groups.

**Figure 2:**
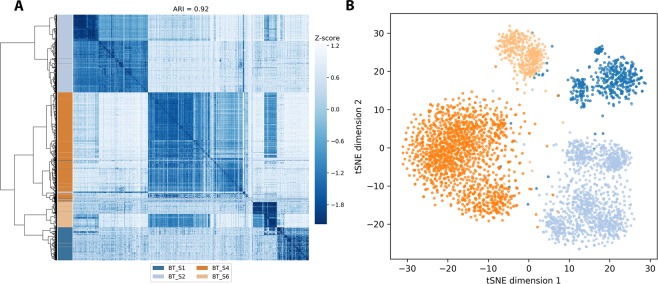
Cluster analysis of the GBM dataset. A representative high-performing (**A**) hierarchical clustering and (**B**) tSNE of the GBM dataset with the same subsets as in Fig. 1 (i.e. excluding variants with MODIFIER impact, variants present in dbSNP and samples with fewer than 50 called variants in total).

Similar results can be seen for the GBM dataset, but the removal of cells based on number of variants has a greater effect on the total number of cells included (Fig. S7); this is likely due to the difference in sequencing depth between the two datasets. GBM clustering results for the same representative subset as for the BC dataset is shown in Fig. 2 for comparison. These results shows that a genotype-centric variant clustering of scRNA-seq data performs well on different datasets across varying experimental parameters.

### Exploring genetic heterogeneity within tumours

**Figure 3:**
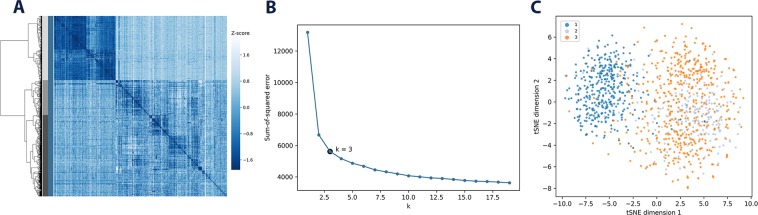
Cluster analysis of the BT_S2 GBM patient. Intra-patient clustering for patient BT_S2, the least heterogeneous GBM patient: (**A**) hierarchical clustering with individual cells coloured shades of grey for the three different clusters, (**B**) SSE-curve with chosen $k=3$, (**C**) a tSNE-visualisation of the clusters.

Given the larger number of cells per patient in the GBM dataset we sought to investigate whether scRNA-seq genotype-centric variant clustering could also be used to find intra-patient genetic heterogeneity. We thus applied the same subsetting criteria as above on a per-patient basis, except with a stricter total variants threshold of 1000 in order to remove cells with variants far below the dataset’s median. The optimal number of *k* clusters for each patient was selected by performing iterative k-means clusterings for $1\le k\le 20$, evaluating each clustering by its sum-of-squared errors (SSE). The optimum was chosen to be $k=4$ in all patients except BT_S2, where $k=3$ was selected. A heatmap, SSE-curve and tSNE for the least heterogeneous patient BT_S2 is shown in Fig. 3 (see Fig. S11 for patient BT_S1, BT_S4 and BT_S6). While this analysis yields three clusters, Fig. 3A,C shows that clusters 2 and 3 are more closely related to each other, while cluster 1 is clearly demarcated from both. Similar results where found for the other patients: some clusters are closely related to others in groups of two, but still separate well to others. Patient BT_S4 yields the least clear results, where all clusters are more closely related to each other than for any other patient.

In order to evaluate the expressed genetic heterogeneity between these intra-patient cell populations, their respective median similarity scores were calculated as previously. Table 2 shows varying degrees of heterogeneity across the patients and their clusters; patient BT_S2 is the least heterogeneous with both the highest and least varying similarity score across its three clusters, as expected. While having an overall higher level of heterogeneity, there is at least one cluster in each of the other patients that reaches close to the same level of stability (*e*.*g*. cluster 1 in patient BT_S6). There are, however, large variations between the different clusters for all patients except BT_S2: for example, cluster 1 in patient BT_S1 has a similarity score of 0.767, while cluster 4 only reaches 0.526.

**Table 2:** Genetic heterogeneity in the GBM dataset across patients and clusters.

Patient	Cluster	Number of cells	Median similarity score
BT_S1	1	55	0.767
	2	46	0.647
	3	32	0.625
	4	334	0.526
BT_S2	1	382	0.847
	2	187	0.848
	3	507	0.828
BT_S4	1	369	0.583
	2	588	0.792
	3	41	0.400
	4	319	0.688
BT_S6	1	17	0.545
	2	135	0.667
	3	111	0.854
	4	76	0.778

In summary, these results demonstrate how genotype-centric scRNA-seq clustering analyses can yield information on genetic heterogeneity in sub-populations of the same tumour.

### Analysis of variant impacts and cell type distributions

It is important to not only explore the varying degrees of genetic heterogeneity present in tumours and their sub-populations, but also within which genes and functional categories this heterogeneity exists. To this end, all variants, their genotypes and their affected genes for each cluster were collected into single, cluster-specific aggregate SNV profiles; only variants that occurred at least twice were included, in order to select cluster-specific variants with high confidence. Each variant in the aggregate profiles were classified into two groups: those that held the exact same genotypes (*e*.*g*. A/G) across all cells in the cluster (“match”), and those that had at least one cell with an alternate genotype (*e*.*g*. A/G in some cells and A/A in others; “mismatch”). Gene lists for each aggregate profile were created by extracting the genes that had at least one matching variant, which were subsequently used for Gene Ontology (GO) enrichment analyses. The top ten terms for each patient and cluster are shown in Figs S12 through S15 and the full lists are included in File S7. A number of patterns can be seen, particularly for immune-related terms (such as neutrophile activation or immune-response activated signalling transduction). There is at least one cluster with several highly enriched such immune-related GO-terms for each patient, but patient BT_S4 stands out: three out of its four clusters are highly enriched for immune-related functions.

**Figure 4:**
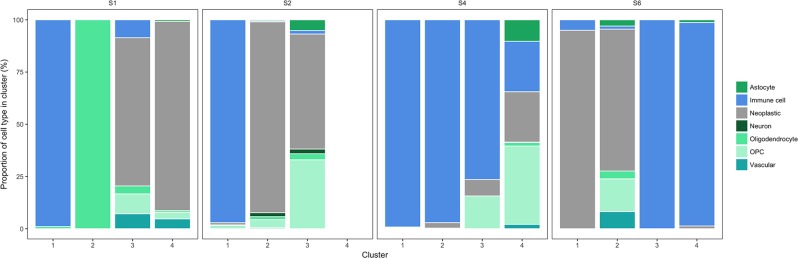
Cell type distributions in each patient sub-cluster. All patients except BT_S2 have three separate sub-clusters (x-axis), and the proportions of each cell type (y-axis) are shown as different colours defined in the legend.

An important aspect of cancer heterogeneity is what cell types are present within individual tumours, which is commonly investigated using cell-specific gene expression markers. The GBM dataset includes such cell type annotations, which may be used to classify the clusters resulting from the variant analysis. Figure 4 highlights immune cell clusters corresponding to the enrichment analyses and that those clusters contain immune cells almost exclusively. Indeed, 9 out of 15 of the variant-based clusters contain a vast majority of a single cell type; only a single cluster have roughly equal proportions of several cell types (cluster 4 in patient BT_S4). Patients BT_S1, BT_S2 and BT_S6 contain at least one cluster containing mostly cells classified as “neoplastic”.

The aggregate profile for each cluster can also be used for several other analyses, such as the variant impact distributions: an impact-specific partition of the genetic heterogeneity may be estimated by calculating the proportions of matched and mismatched variants for each cluster. There is an overall higher proportion of mismatched HIGH and MODERATE impact variants compared to the lower impacts, while also having fewer high-impact variants overall (Fig. S16); this is in line with what was shown for the BC dataset.

In summary, these results demonstrate how genetic heterogeneity at the RNA-level may be used to investigate the impact of protein functions within sub-clusters, and that variant-based cell-type distributions correspond well to gene expression-based classifications.

### Genetic heterogeneity reveal novel variants in glioblastoma patients

**Figure 5:**
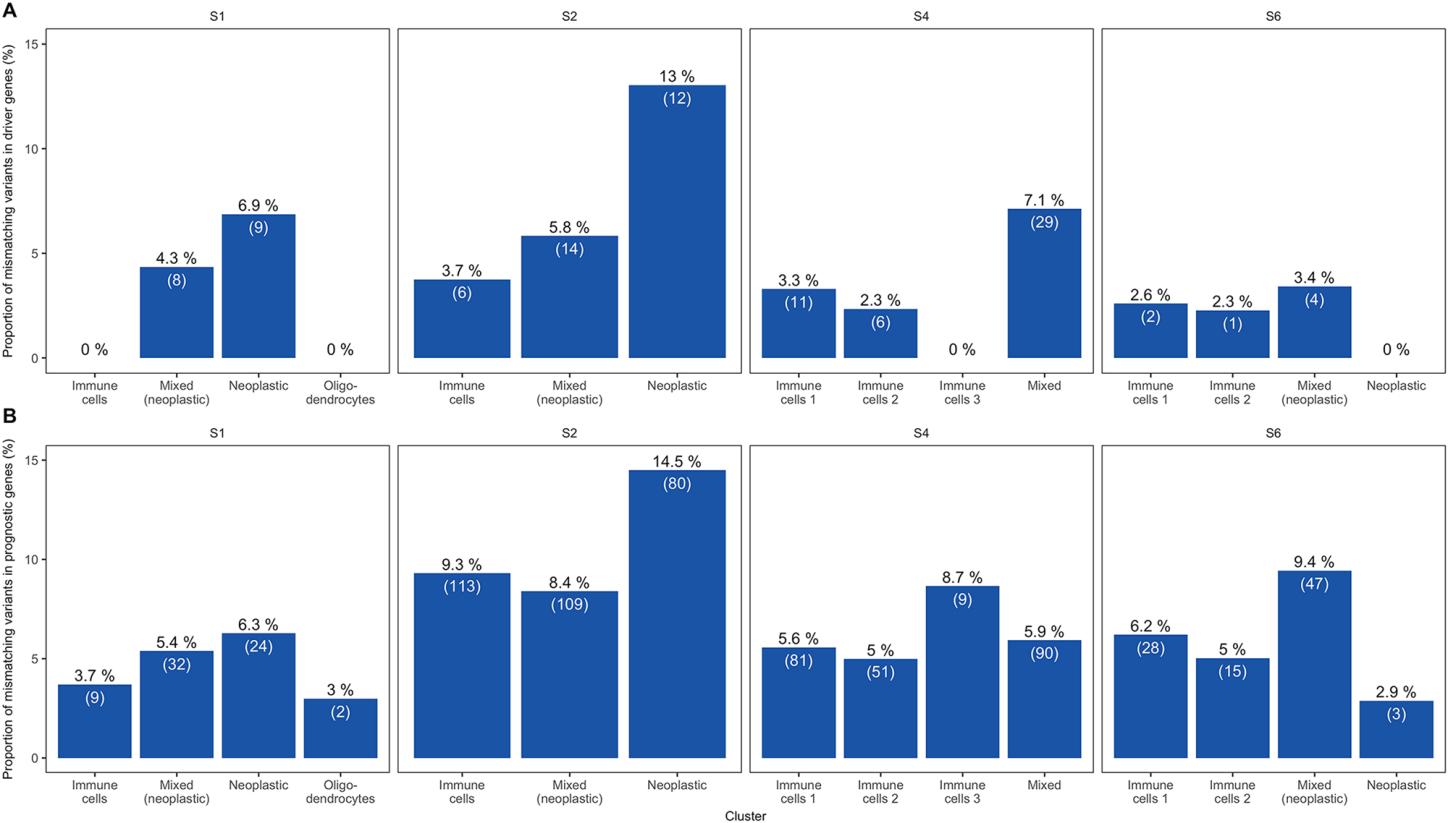
Analyses of known driver and prognostic marker genes. Proportions of variants with at least one mismatch in the aggregate profiles for each patient sub-cluster and known driver genes (**A**) and prognostic marker genes (**B**).

An important consideration for tumour evolution in terms of therapeutic effectiveness is that of driver mutations: some mutations effectively close off some targeted therapies for patients possessing them. While bulk sequencing experiments can give information on the tumour as a whole and guide therapy, if only a single cell contains a driver mutation that inhibits an attempted therapy a relapse at a later time is highly likely, which represents a big problem in today’s clinics. We thus sought to investigate the number of driver gene variants in the GBM clusters by using the aggregate profiles. The *IntOGen* mutations platforms contains driver genes for several cancer types, including glioblastoma, which were used for this analysis^33^. The proportion of mismatched variants in driver genes are the highest for the clusters containing predominantly neoplastic cells, followed by mixed clusters with a large proportion of neoplastic cells (Fig. 5A). The only exception to this is the neoplastic cluster in patient BT_S6, which only contains 17 cells in total. All immune cell clusters show either 0 or below 4% mismatches. There is, however, no patient where all non-neoplastic clusters show 0% mismatch, highlighting the extent of intra-tumour heterogeneity and the importance of single cell analyses.

There are also other types of important genes, such as the prognostic markers from the *Pathology Atlas*^34^. Variants in these genes were analysed in the same manner as the driver genes above and visualised in Fig. 5B. The proportion of mismatching variants in these prognostic marker genes show the same general patterns as for the driver genes (*i*.*e*. more mismatches for neoplastic clusters), with a difference being that the third immune cell cluster of patient BT_S4 now shows the highest proportion of mismatches for that patient (it does, however, contain ten times fewer variants that the other clusters).

While analyses of already known cancer-related variants and their affected genes are important, the discovery of novel variants holds the potential to further increase and deepen the collective knowledge concerning cancer and its effects. We thus sought to evaluate if there are any variants common across the three distinct neoplastic clusters found for patients BT_S1, BT_S2 and BT_S6. To this end we compared the aggregated profiles across the neoplastic clusters subset to only include missense variants, in order to focus on mutations likely to have an effect on cellular functions. For variants where several genotypes were available in a cluster only the most common one was included.

A total of 166 common variants across the three patients were found. Of these were 163 already known variants present in *dbSNP,* indicating that the general discovery strategy is sound, in addition to three previously unknown variants. Interestingly, the *rs2853508* variant in the cytochrome b gene (MT-CYB) has previously been shown to likely be pathogenic in breast cancer. While this particular variant has not been shown to be present in glioblastoma, there are a number of other MT-CYB variants that have^35^. The mean expression of MT-CYB across the three neoplastic clusters is 3918 TPM, *i*.*e*. highly expressed. The rs2853508 variant may thus also play a hitherto unexplored role in glioblastoma, but this would need to be further investigated. While the MT-CYB gene is the most highly expressed gene found to be affected by the common variants, there are also 36 other genes that have a mean TPM above 100 (none of the 163 known variants have a mean TPM below 1). These variants are thus likely to be expressed at the protein level and may affect their overall function.

The three novel variants affect three different genes: HLA-F, SLC38A2 and TBCD (Table S5). The genotypes for these variants are matches across all neoplastic clusters, with the exception of HLA-F in patient BT_S6 (G/G in patients BT_S1 and BT_S2 but G/A in BT_S6). Given the presence of these three exact same variants in three different glioblastoma patients, they constitute possible avenues for further targeted studies and may prove important in the general mutational landscape of glioblastoma. It must be noted, however, that the number of patients in the GBM dataset likely is too small to definitively conclude this, but that the larger number of common previously known variants highlights the soundness of the general methodology.

In summary, these results demonstrate how scRNA-seq variant clustering can be used to analyse both known variants as well as discover unknown potential candidates for future studies.

## Discussion

RNA-seq analyses commonly focus on gene expression and conclusions derived thereof, leaving a large part of the data unused, *i*.*e*. the sequence information. We have previously shown the potential of using this sequence information for variant analysis as a tool for cell line authentication^25^ and investigations of genetic heterogeneity expressed at the RNA-level^26,27^ in bulk RNA-seq data, but this has yet to be applied at the single-cell level. In order to evaluate this possibility, we applied the previously established methodology on two separate scRNA-seq datasets containing patients with breast cancer and glioblastoma^17,18^. The analyses presented herein differs from other single-cell methods in that it takes the genotypes of each individual variant into account (rather than merely the presence or absence of variants in each cell), in addition to not directly including gene expression information. This allows for in-depth analysis of pure genetic variation and its impact on protein function between cells at the single-base level, fully accounting for differences between variant genotypes.

The inter-patient heterogeneity between breast cancer patients highlighted by our analysis is corroborated by well-established knowledge of the disease^36^. The analysis of impact-specific heterogeneity show that higher-impact variants contribute more to the heterogeneity than lower-impact variants; this is most pronounced for the HIGH impact category. This likely reflects the different stages of tumour evolution within patients, where the higher-impact variants bestow a selective growth advantage for cells in which they occur^2^. Interestingly, the metastases show statistically significant lower levels of overall genetic heterogeneity compared to their corresponding core tumours. This holds true for the higher-impact variant categories as well, while higher levels of heterogeneity in metastases were found for the lower-impact categories.

Previous studies have shown conflicting data regarding genetic heterogeneity in breast cancer metastases: one concluded that most driver mutations (*i*.*e*. higher-impact mutations) are conserved from the core tumour into the metastasis^37^, while another showed that metastases have a higher level of mutational burden for previously established cancer-related genes^38^. As both of these studies were conducted on bulk sequencing data they would likely have missed cell-specific variants and, subsequently, a portion of intra-tumour heterogeneity. Our results indicate that metastases generally have a lower degree of genetic heterogeneity in higher-impact variants than their corresponding core tumour. It should be noted that this is for a small cohort, and further single-cell studies would need to be conducted in order to fully investigate this. While the breast cancer subtype-specific heterogeneity shown here is both statistically significant and corroborated by previous studies, the same sample size limitation apply^36^. The results do indicate, however, that the general methodology is both sound and highly useful for investigating single-cell data.

The same type of inter-patient heterogeneity was also found between the glioblastoma patients, but at a higher level than for the BC dataset. While the difference in median similarity score between the most and least heterogeneous BC patients was 0.135, the corresponding number for the GBM dataset was 0.357. This difference is likely explained by the established knowledge that GBM is among the most heterogeneous and deadly of all human cancers^39–41^.

Cluster analysis of a previously published bulk RNA-seq dataset as an initial test yielded a perfect ARI of 1, indicating the general power of the method, but the non-optimised results of the single cell clusterings did not perform as perfectly. The BC clustering yielded an ARI of 0.51, while corresponding number for the GBM dataset is 0.86; while not as impressive as for bulk data, these results demonstrate that the methodology holds great discriminative power for single cell data as well. The difference between these performances is likely due to the nature of the datasets: the BC data contains fewer cells per patient (around 50), while the GBM dataset has an order of magnitude more (above 800). This may be an effect of the general stochasticity seen in single cell data^42^; the discriminating power of cluster analyses may increase with the number of cells per group, as more data is available for distinguishing and separating each group.

The evaluations of different variant subsets demonstrate that the number of cells per group is not the only important parameter, however: the variants’ putative impact on protein function and the presence/absence in dbSNP were informative parameters for both datasets, yielding performances above 0.95 ARI. The increase in performance gained from removing MODIFIER-impact variants may be due to higher levels of noise: as these variants likely have little to no effect on cells, their mutational patterns will be more random than for higher impact variants. A possible explanation for the higher performance of the similarity score compared to the other metrics employed is its use of a weighted number of matching genotypes, which could be especially suited to the stoachastic nature of single-cell data. Other scRNA-seq clustering methods based on gene expression of cell line data have yielded ARIs between 0.15 and 0.91^16^. These results demonstrate that scRNA-seq variant analysis is a powerful method for clustering both bulk and single cell RNA-seq data.

The intra-patient sub-clusters of the GBM dataset demonstrate how scRNA-seq variant analysis can be used to separate a tumour into distinct sub-populations. Analysis of variant-harbouring genes revealed several functional categories as being differentially enriched in the clusters, which were corroborated by cell type annotations from the original study^18^. While a majority of the clusters predominantly consisted of a single cell type, the varying degrees of cluster purity highlight the complexities of intra-tumour heterogeneity. The genetic heterogeneity displayed in each cluster demonstrates that a variant-based analysis corresponds well to expression-based ones (such as the cell type-determination performed in the original GBM study), but that a part of the heterogeneity within tumours is lost when solely examining gene expression. Variant analysis of scRNA-seq data thus represents a useful complement to the already established gene expression analyses.

The impact distributions of the GBM clusters mirror the results from the BC dataset, *i*.*e*. that there is a larger within-cluster variation of higher-impact variants than for lower impacts. While the global analyses of variant impacts yields information on cellular heterogeneity across functional categories as a whole, the analysis of variants within driver and prognostic genes can highlight mutational effects in previously known and well-established cancer contexts. While a general heterogeneity of these genes can be seen within most patient sub-clusters, it is most pronounced for the neoplastic clusters.

The analysis of variants common across the three distinct neoplastic intra-patient clusters highlights three novel variants in three separate genes that have yet to be described, in addition to 163 already described variants. HLA-F is a non-classical MHC-class I gene, the function of which is relatively unknown and less studied than other MHC molecules^43^. It has been shown to bind calreticulin and TAP, in addition to likely only interact with a small number of peptides^44^. HLA-F has also been implicated in gastric cancer, along with another of its family, HLA-E^45^. The SLC38A2 gene (also known as SNAT2) is a sodium-dependent amino acid transporter^46^. It is widely expressed in the central nervous system and has been shown to activate the mTOR signalling pathway, an important network for cancer progression^47–49^. TBCD is one of five cofactors required for tubulin complex assembly^50^. Microtubules are important for the cytoskeleton, transporting cargo and neuronal morphology, and mutations in these cofactors are implicated in neurodegenerative disorders^51,52^. Several TBCD variants have been described for *e*.*g*. microencephalopathy^53^.

The number of variants present in the COSMIC database for these three genes are 68 (HLA-F), 119 (SLC38A2) and 123 (TBCD), highlighting that these genes are already implicated in cancer^54^. The novel variants found in this scRNA-seq dataset might thus constitute previously unknown but relevant mutations for glioblastoma. Among the already known variants is the rs2853508 mutation in the MT-CYB gene, which may have a pathogenic effect in breast cancer according to dbSNP. Its presence in the three neoplastic clusters may thus also implicate it to be of relevance for glioblastoma patients. The number of patients available in this dataset is small, however, and more in-depth studies would be needed to verify the importance of these mutations.

Given these results, genotype-centric analysis of genetic heterogeneity at the RNA-level may represent a highly useful tool that can be used in conjunction with already existing methods to further explore and analyse RNA-seq data. Indeed, it is conceivable that RNA-seq variant analysis can be used instead of genetic sequencing methods in order to explore expressed mutations routinely, a notion supported by several variant-level RNA-seq studies^19–21,23,31,32^. While this would only allow for analysis of the expressed variants, it opens up the possibility of applying multi-omics approaches^55^ to single cell data that contains both variants, gene expression and *e*.*g*. protein expression. Such an analysis would allow an unprecedented coverage of a wide array of biological aspects in single cell analysis and greatly increase its utility. While several strategies for analysing both DNA and RNA from single cells have already been proposed, they are limited to analyse only DNA/RNA^56,57^. Using RNA-seq variants could thus cover a wider array of molecules (*e*.*g*. DNA, RNA and protein), if the main interest is in expressed variants only.

In summary, we have applied a genotype-centric analysis of genetic heterogeneity on two separate scRNA-seq datasets and shown that such analyses represent a powerful and versatile complement to already existing computational tools. The main findings include the presence of inter- and intra-patient heterogeneity in both datasets, where variants predicted to have an high to moderate impact on protein function were shown to have a larger degree of heterogeneity than lower-impact variants. Metastatic breast cancer lesions were also demonstrated to have a lower level of genetic heterogeneity compared to their corresponding core tumours. Furthermore, the results show that variant analyses can separate scRNA-seq data into patient-specific sub-clusters that correspond well to different cell types and indicate that neoplastic clusters have a generally higher level of heterogeneity than non-neoplastic ones. Additionally, variant-based single-cell clustering generally performs better for datasets with a larger number of cells compared to having higher sequencing depth. Finally, three novel missense SNVs common to all neoplastic GBM clusters across three patients were found in genes previously implicated in cancer and other diseases. These results demonstrate how RNA-based variant analysis may be used in conjuncture with the already well-established gene expression analysis tools, thus greatly increasing the maximal utility of any scRNA-seq dataset. This could prove especially useful in single-cell multi-omics, where RNA-seq variants could be used instead of *e*.*g*. exome sequencing to analyse expressed variants together with both gene and protein expression.

## Methods

### Raw data download and pre-processing

Metadata from the GEO and SRA was extracted using a custom R-script together with the *GEOquery*^58^ and *SRAdb*^59^ packages in addition to the NCBI *E-utilities*^60^. Data from variant calling (such as the number of calls per cell) was added, and free-text fields were manually separated into individual and informative columns (such as patient, location and cell type); the final metadata can be found in File S4–S6. The raw data was downloaded and processed as previously described^25^. In short, FASTQ files were download using the *fastq-dump* utility from the *SRA toolkit*^61^ and subsequently aligned using the *STAR* aligner^28^ in 2-pass mode. Variant calling was performed using the GATK RNA-seq Best Practices^29^ and annotated using snpEff^30^. Gene expression estimations were performed using the *Salmon* software^62^. The GRCh38 human genome assembly was used where applicable.

### Clustering of scRNA-seq variant data

SNV profiles were created for each single cell by excluding variants based on the following criteria: *Fisher strand* ≤ 30, *quality by depth* ≤ 2, containing more than three SNV clusters within a 35 base pair window and, lastly, variants with a sequencing depth lower than ten. Similarity scores (defined as $(s+a)\div(n+a+b)$, where *s* is the number of matching variant genotypes, *n* is the total number of overlapping variant positions, $a=1$ and $b=5$) for the different subsets described in the *Results* section were calculated as previously described for each pairwise single-cell profile comparison within each dataset and stored as distance matrices^26^. These were subsequently used as input for hierarchical agglomerative clustering using Ward linkage, evaluated with the Adjusted Rand Index (ARI) and visualised as heatmaps with dendrograms as well as t-distributed stochastic neighbour embeddings (tSNEs).

Evaluation of the optimal *k* clusters was done with the “elbow method” with sum-of-squared errors (SSE) for iterative k-means clusterings of *k*’s up to 20. The optimal *k* was chosen geometrically: define *l*_1_ as the line whose end points are given by the start and end points of the SSE curve, *p* as the point on the SSE curve for a particular *k* and *l*_2_ as the line perpendicular from *l*_1_ to *p*. The optimal *k* is then chosen as the *k* with the maximum length of *l*_2_, following visual inspection of both the SSE curve and the dendrogram.

### Profile aggregation, enrichment and gene analyses

Enrichment analyses were performed on the aggregate profiles described in the *Results* section using the *clusterProfiler*^63^ R-package using the “GOslim” enrichment category (which removes redundancy in the original GO hierarchy). A total number of 75 driver genes for “glioblastoma multiforme” were downloaded from the *IntOGen*^33^ website. Thirty of these 75 were listed as “known driver genes”, which were subsequently used for the analyses. Prognostic marker genes were downloaded from the *HPA Pathology Atlas*^34^ website and subset for the “glioma” cancer type, which were subsequently used for the analyses.

### Code availability and statistical analyses

The clustering and aggregation analyses were implemented as an open source software package for the Python programming language named *VarClust*, which is available at https://github.com/fasterius/VarClust. Supplementary code for the cluster analyses is available in SCode 1, a Jupyter Notebook document. Code pertaining to all other analyses can be found in SCode 2 (a RMarkdown document), including statistical calculations and visualisations. Two-tailed t-tests were used where applicable and a significance level of $\alpha =0.01$ was used for all statistical tests.

## Supplementary information

Supplementary figures and tables

Supplementary code for data analysis

Supplementary code for generation of figures

Supplementary information on cell lines

Supplementary information on breast cancer

Supplementary information on glioblastoma

Supplementary information Gene Ontology enrichment analysis

## Data Availability

All data used in this paper is publicly available at the GEO.
