# Supplementary material for: Single-cell RNA-seq variant analysis for exploration of genetic heterogeneity in cancer
Source: Sci Rep. 2019 Jul 2;9:9524. doi: 10.1038/s41598-019-45934-1 (PMC6606766; doi:10.1038/s41598-019-45934-1)
Supplement: Supplementary file 1 — Supplementary figures and tables [file 41598_2019_45934_MOESM1_ESM.pdf]

Single-cell RNA-seq variant analysis for exploration of genetic heterogeneity in cancer

Supplementary Information

Erik Fasterius[1], Mathias Uhlén[1,2], and Cristina Al-Khalili Szigyarto[1,2,*]

[1]School of Chemistry, Biotechnology and Health, KTH Royal Institute of Technology, Stockholm, Sweden
[2]Science for Life Laboratory, KTH Royal Institute of Technology, Solna, Sweden
[*]Corresponding author: *caks@kth.se*

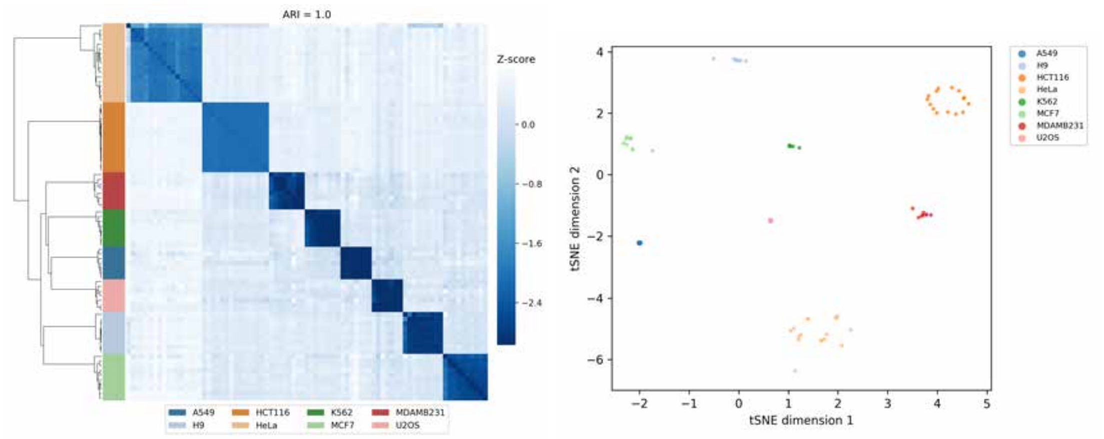

Supplementary Figure 1: Hierarchical clustering (left) and tSNE visualisation (right) of the previously described publicly available bulk cell line cohort.

Supplementary Table 1: Sequencing parameters for the two scRNA-seq datasets.

Dataset	Read layout	Number of patients	Median cells per patient	Median SNVs per cell
Breast cancer	100×2	11	50	9113
Glioblastoma	75×2	4	829	1997

Supplementary Table 2: Inter-patient heterogeneity for the GBM dataset.

Patient	Similarity score	Cells
BT_S1	0.471	467
BT_S2	0.828	1076
BT_S4	0.621	1317
BT_S6	0.636	339

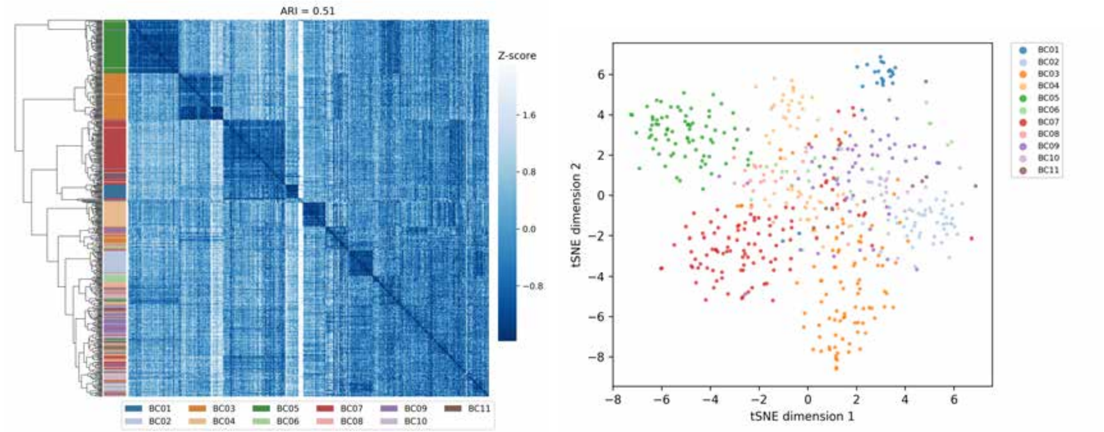

Supplementary Figure 2: Hierarchical clustering of the BC datasets with default parameters and no variant subsets.

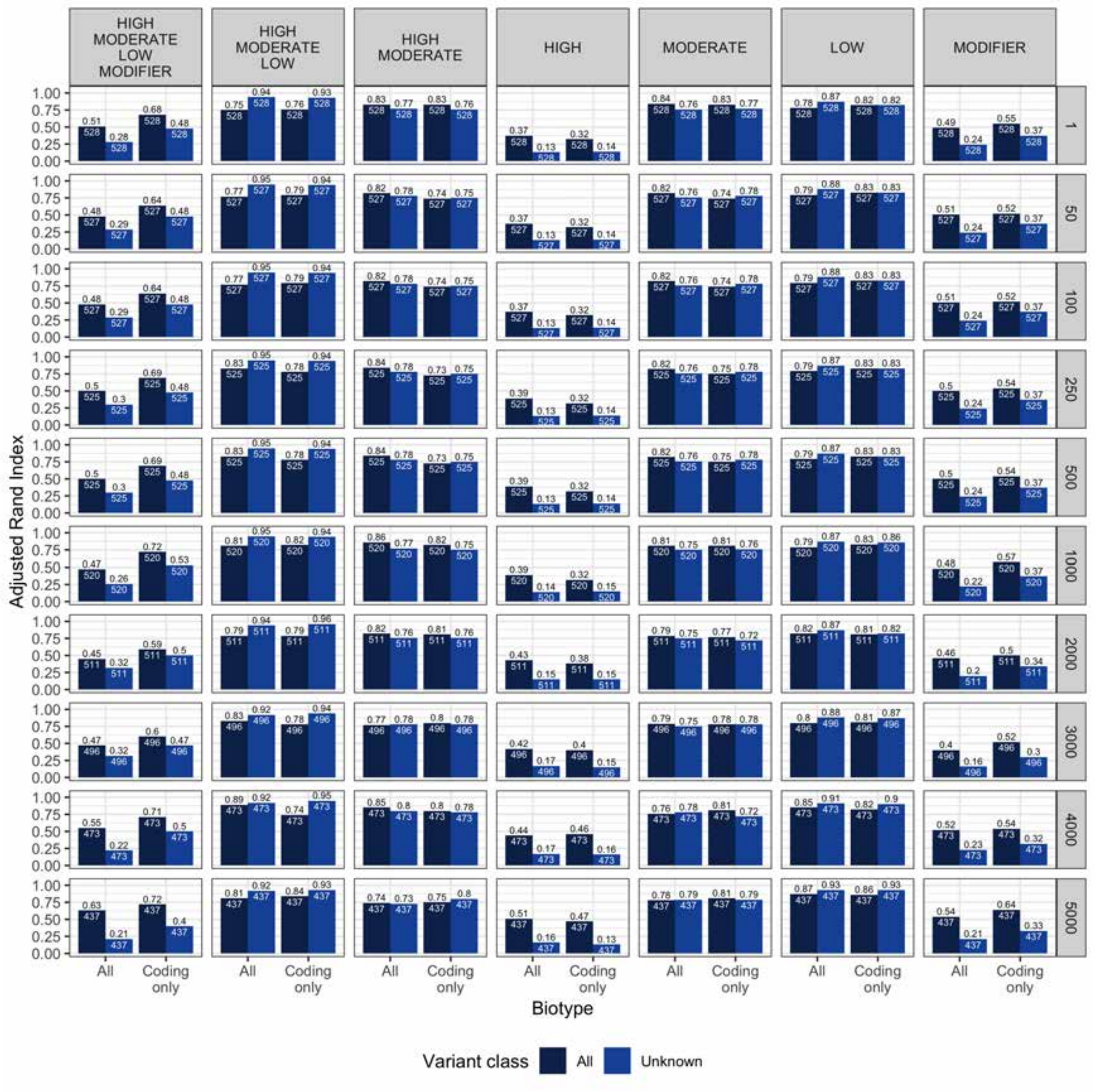

Supplementary Figure 3: Evaluation of different variant subsets for clustering the BC dataset with similarity score.

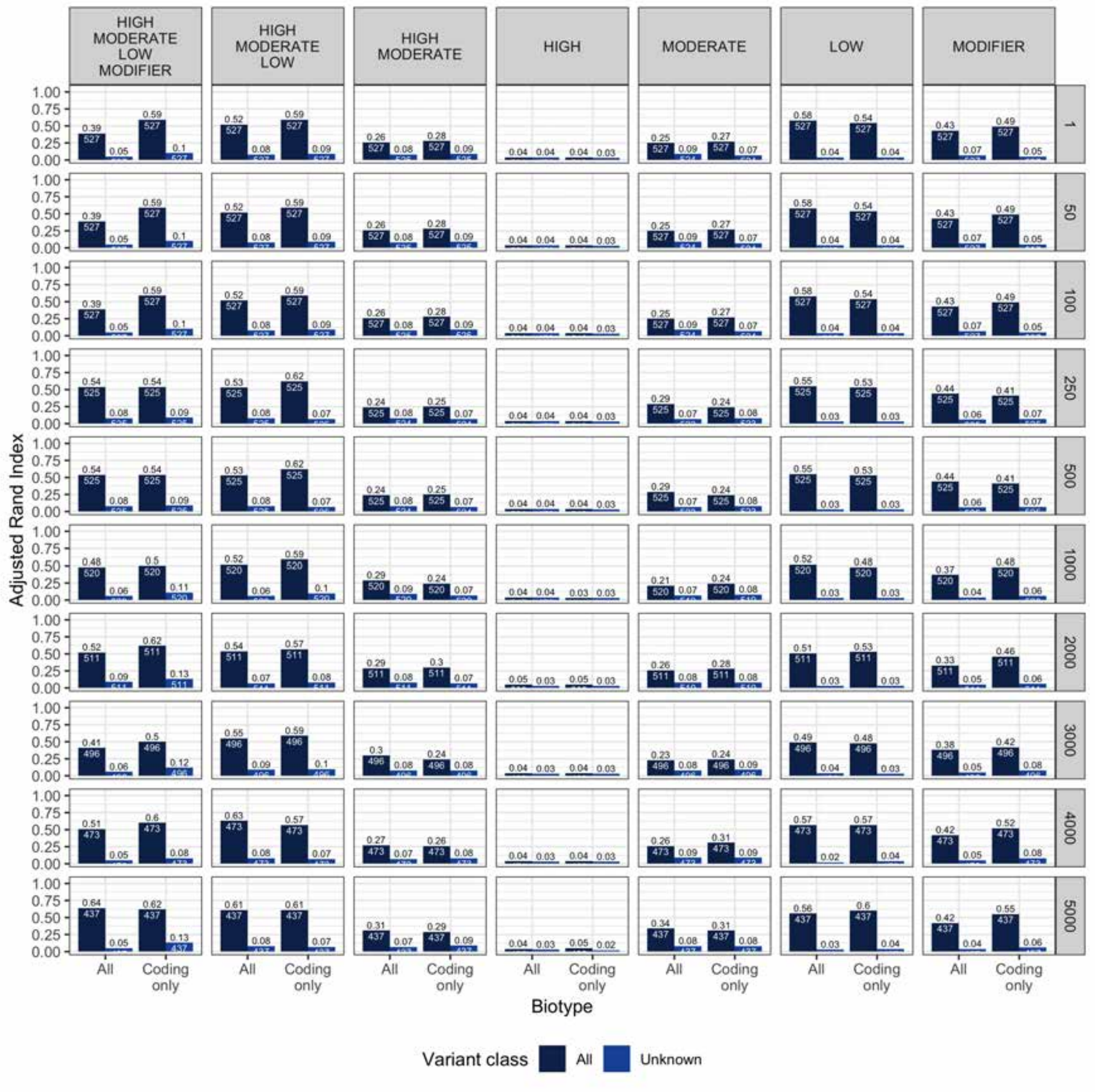

Supplementary Figure 4: Evaluation of different variant subsets for clustering the BC dataset with concordance.

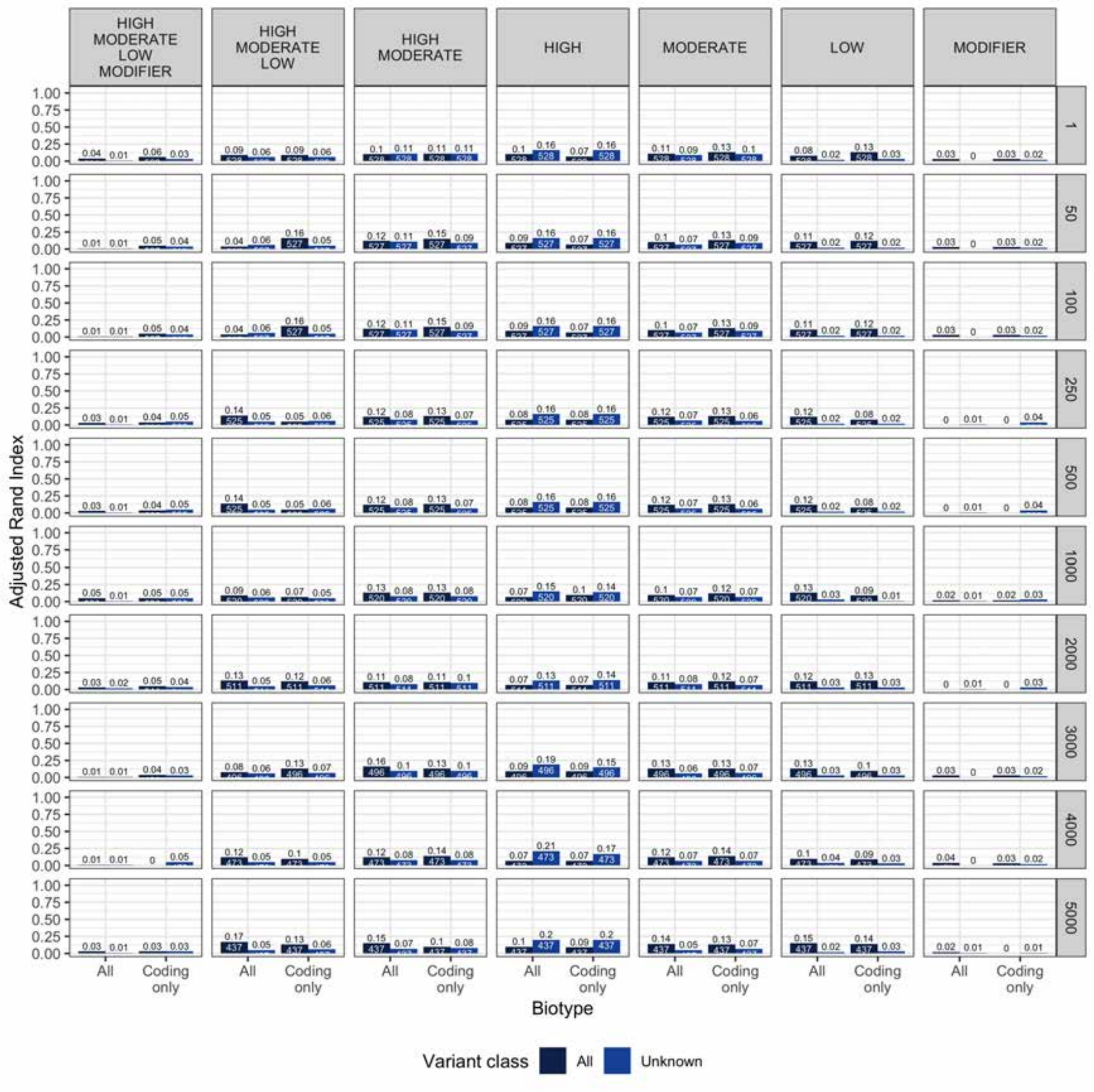

Supplementary Figure 5: Evaluation of different variant subsets for clustering the BC dataset with Hamming distance.

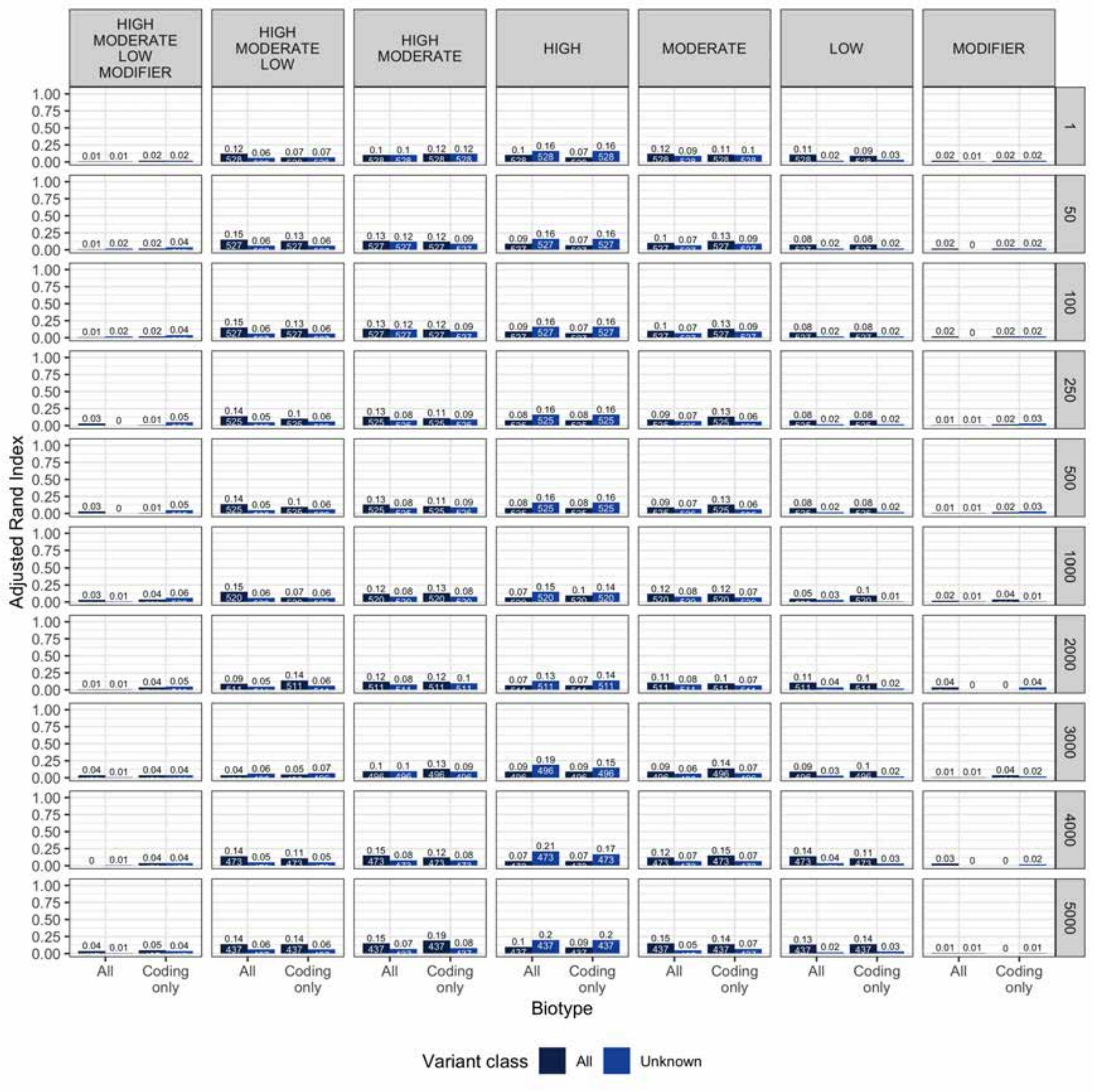

Supplementary Figure 6: Evaluation of different variant subsets for clustering the BC dataset with Levenshtein distance (also known as *edit distance*).

Supplementary Table 3: Average variants for each cell in the BC dataset across different categories.

Impact subset	Average number of variants
HIGH	235
MODERATE	1877
LOW	6893
MODIFIER	39408
Class subset	Average number of variants
Known	28193
Unknown	20222

Supplementary Table 4: Genetic heterogeneity across cancer subtypes in the BC dataset.

Subtype	Median similarity score	Number of cells	Number of patients
ER+	0.696	57	2
ER+/HER2+	0.700	33	1
HER2+	0.688	94	3
TNBC	0.684	79	5

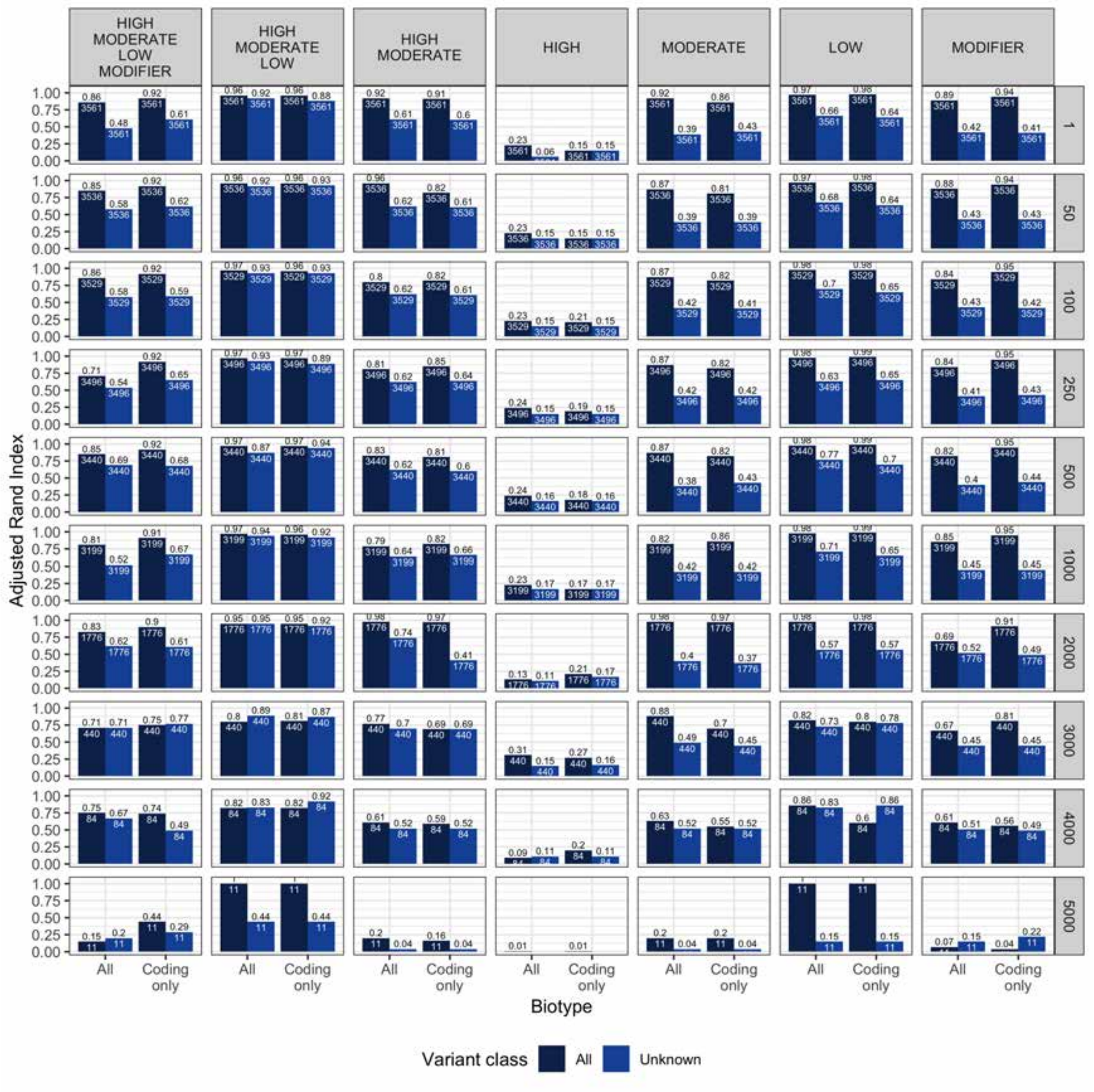

Supplementary Figure 7: Evaluation of different variant subsets for clustering the GBM dataset with similarity score.

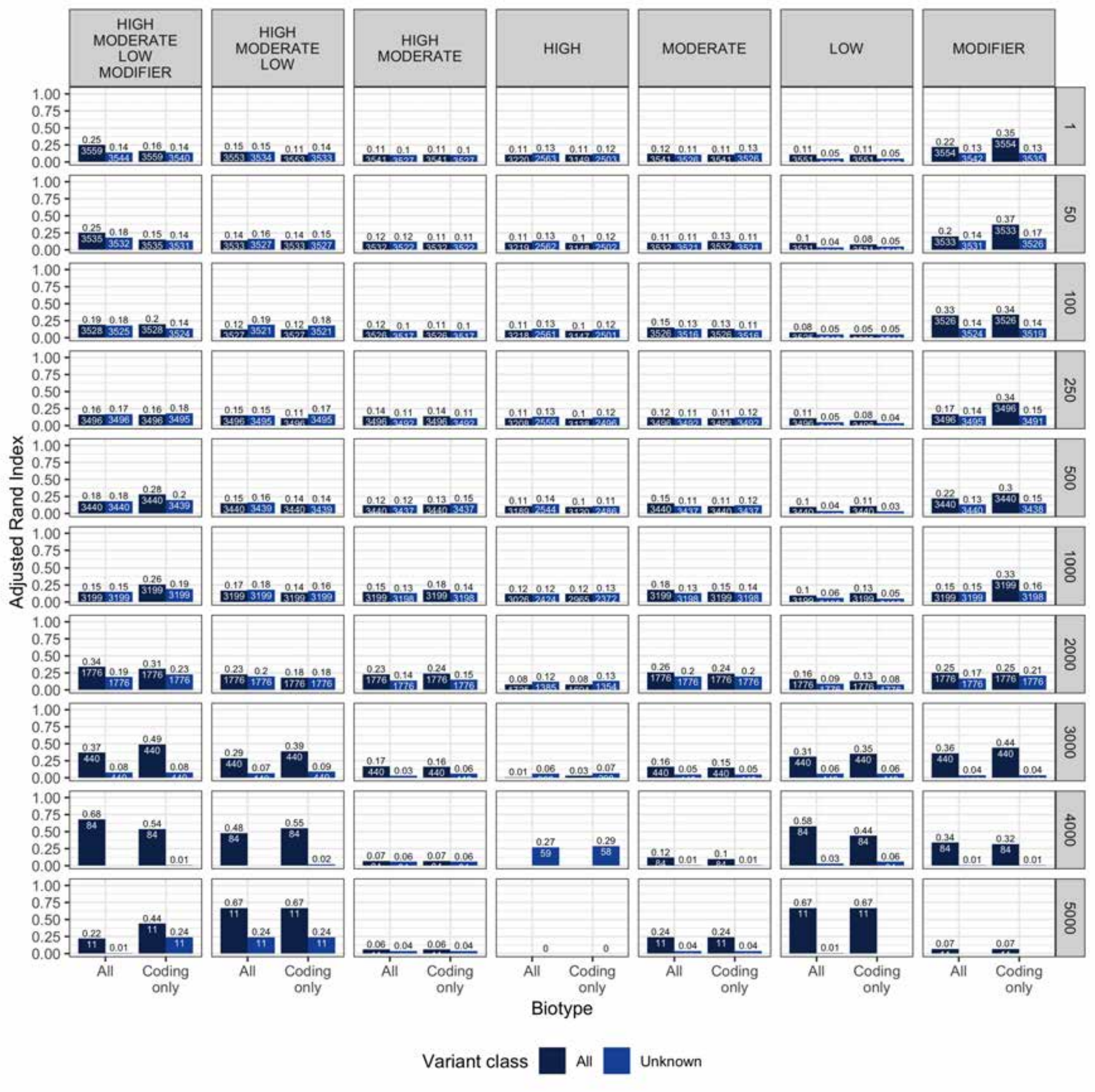

Supplementary Figure 8: Evaluation of different variant subsets for clustering the GBM dataset with concordance.

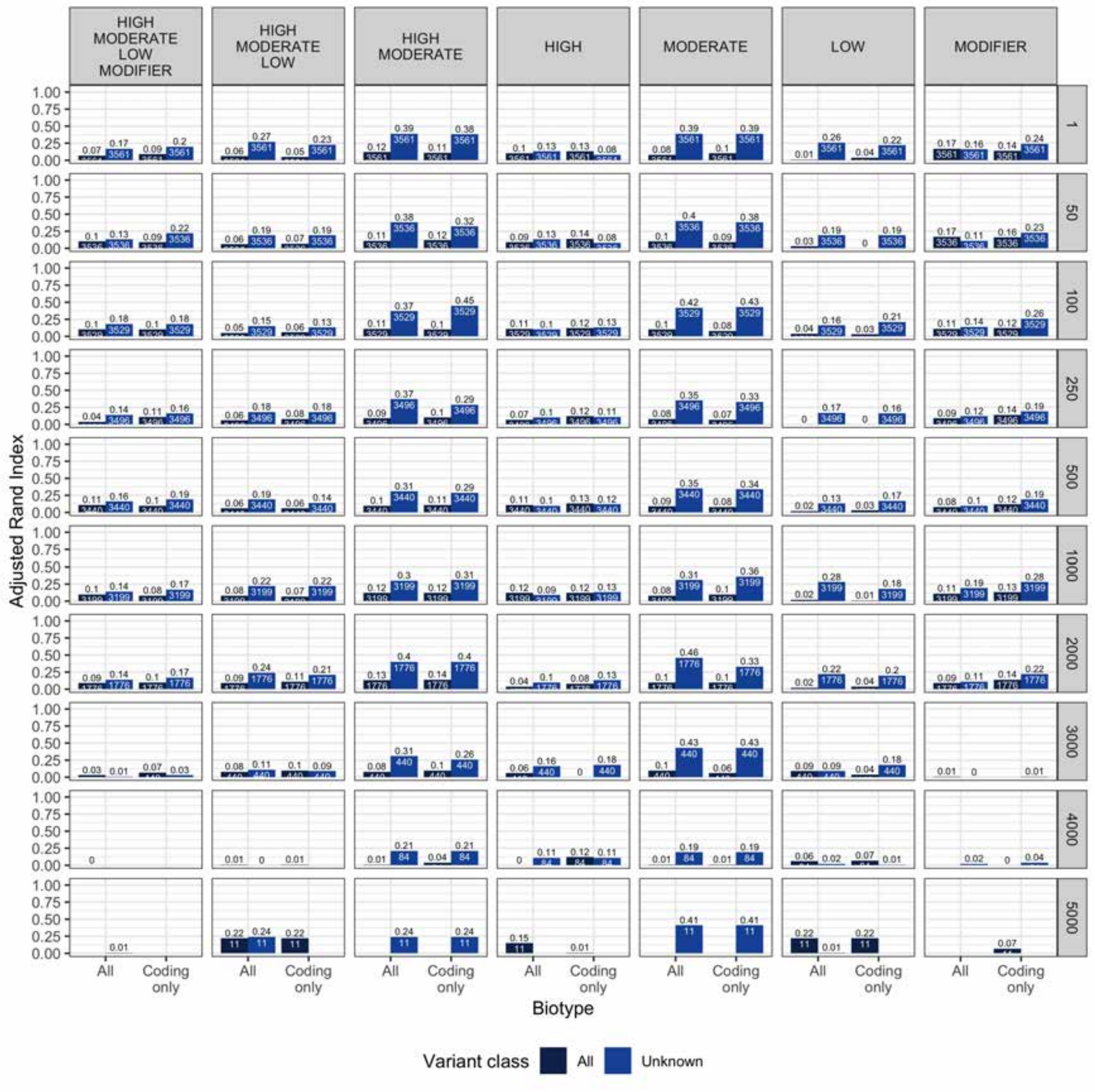

Supplementary Figure 9: Evaluation of different variant subsets for clustering the GBM dataset with Hamming distance.

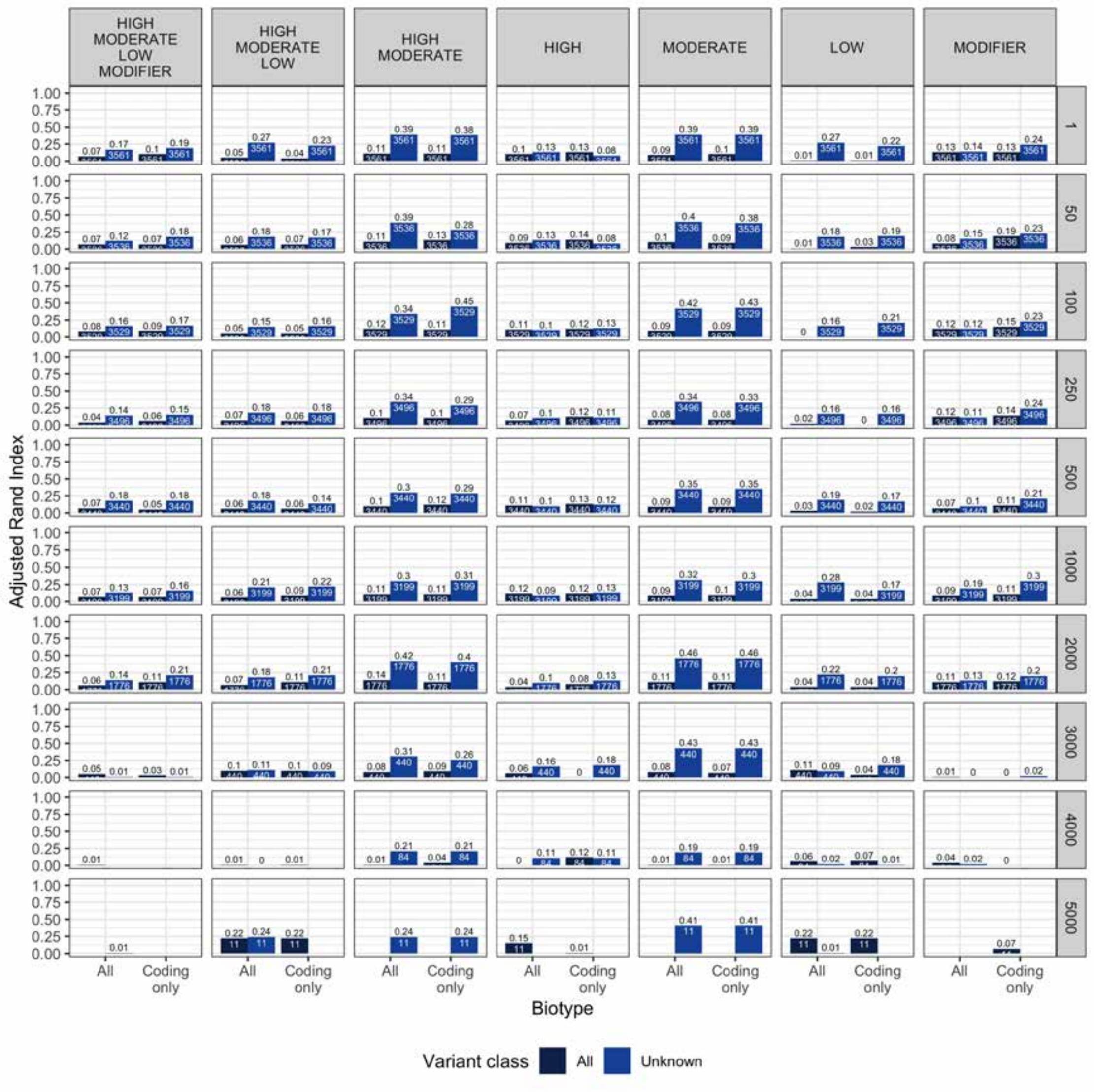

Supplementary Figure 10: Evaluation of different variant subsets for clustering the GBM dataset with Levenshtein distance (also known as *edit distance*).

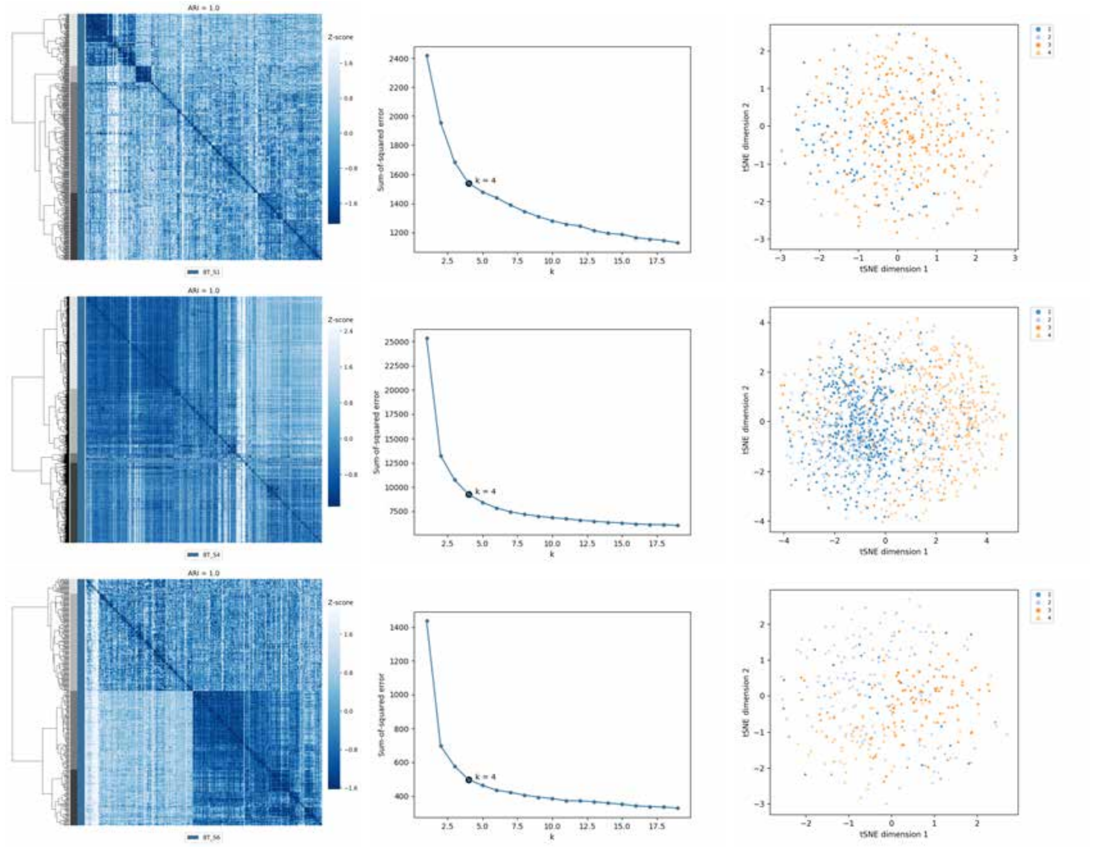

Supplementary Figure 11: Intra-patient clustering for patients BT_S1, BT_S4 and BT_S6.

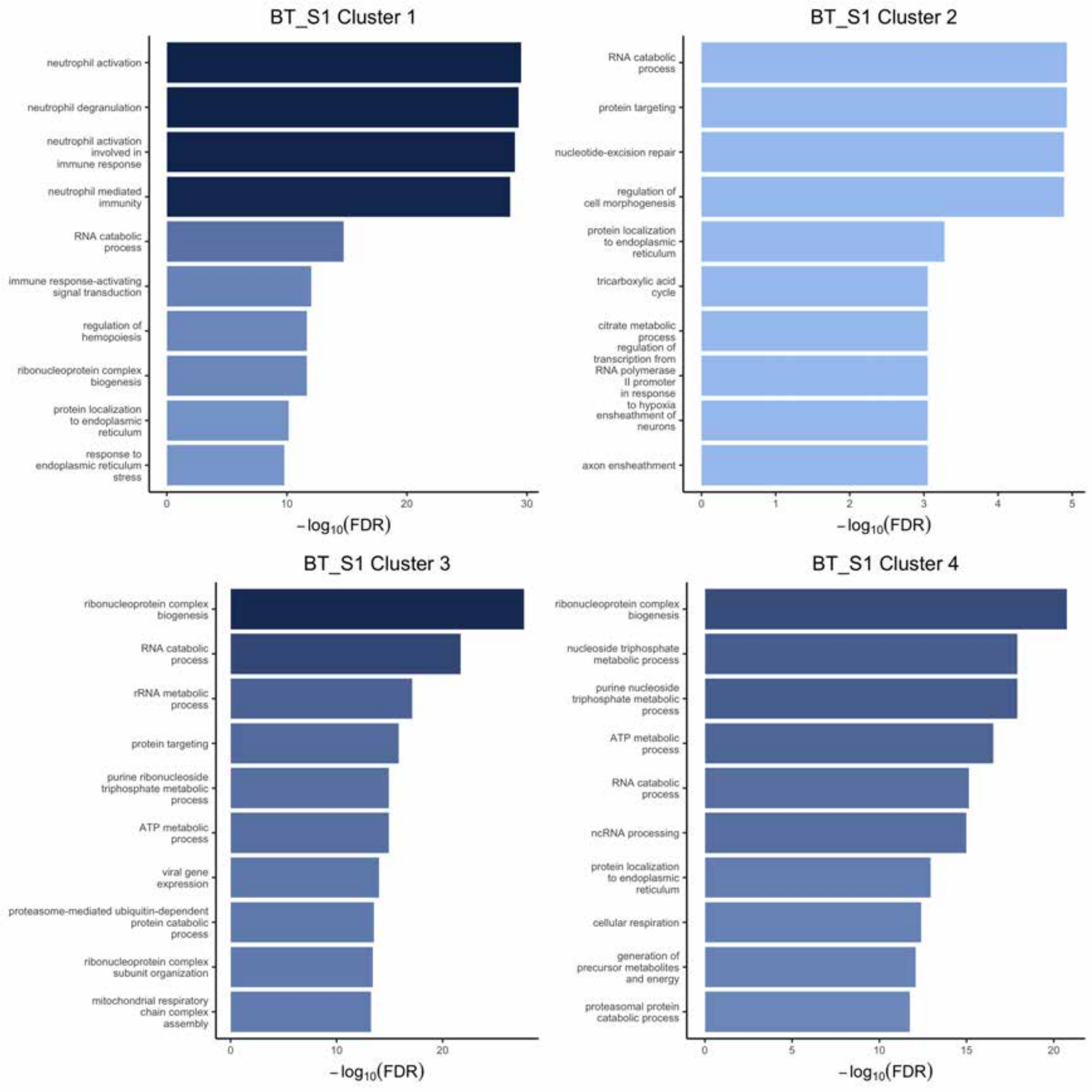

Supplementary Figure 12: Top 10 enriched GO-terms for the BT_S1 patient clusters.

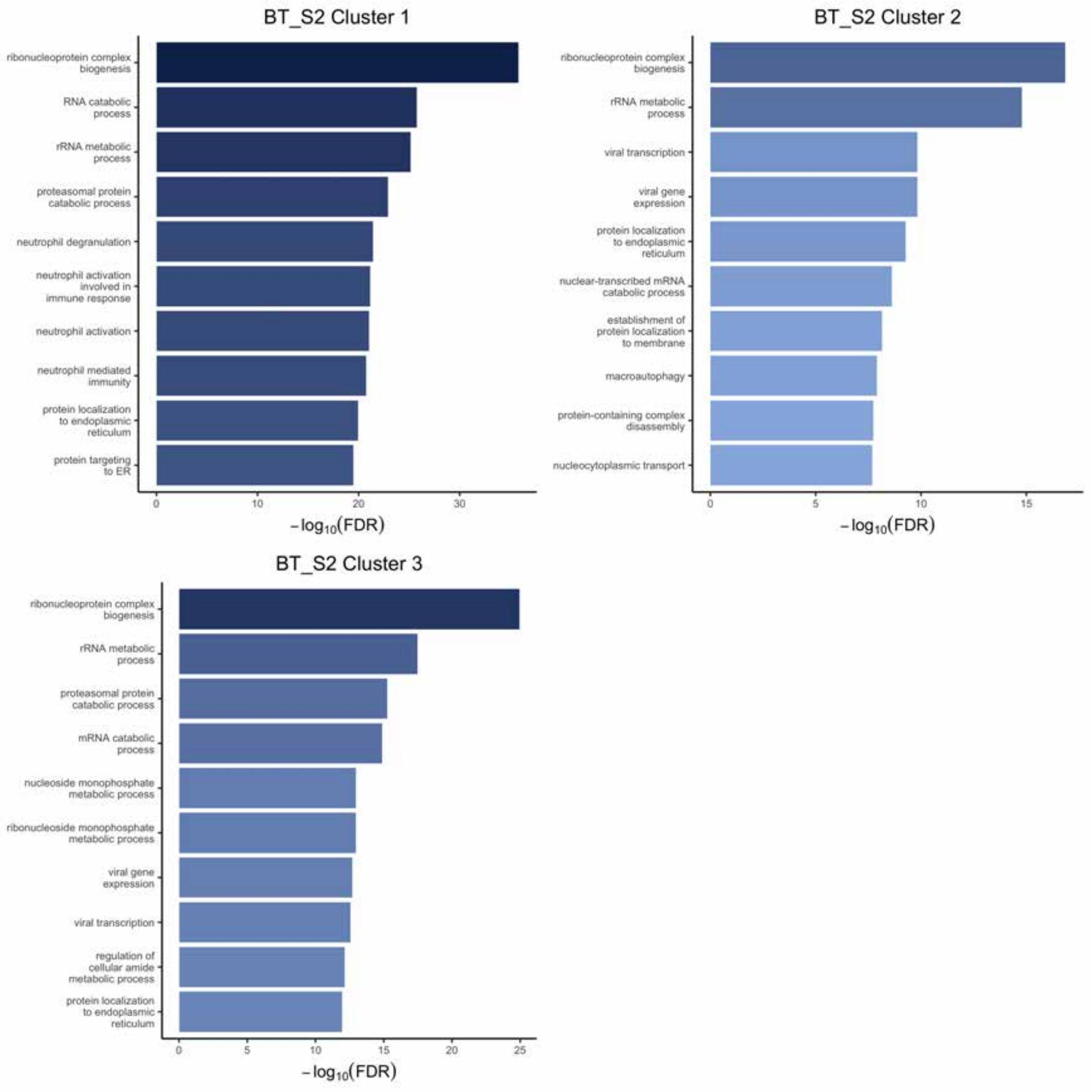

Supplementary Figure 13: Top 10 enriched GO-terms for the BT_S2 patient clusters.

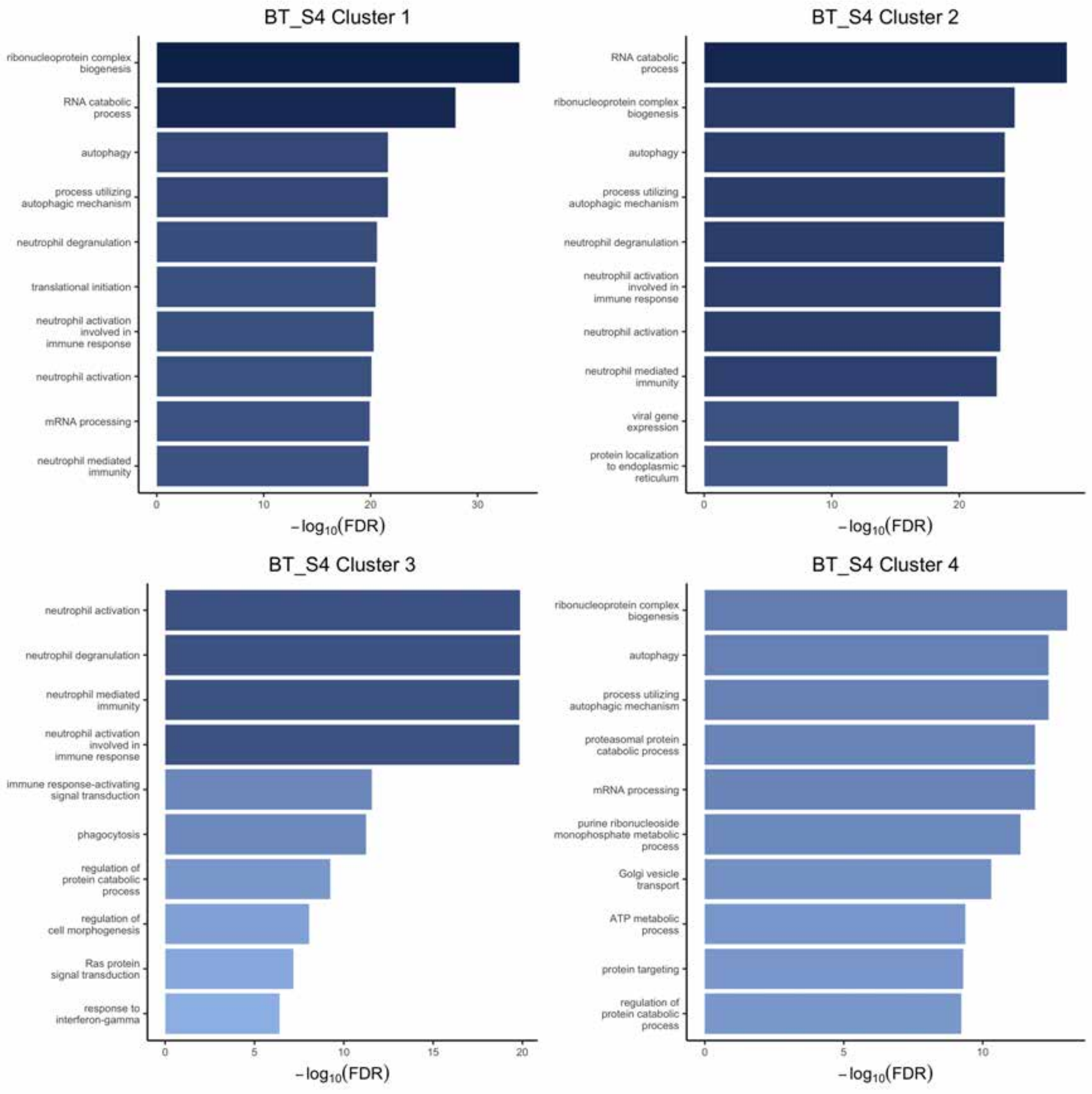

Supplementary Figure 14: Top 10 enriched GO-terms for the BT_S4 patient clusters.

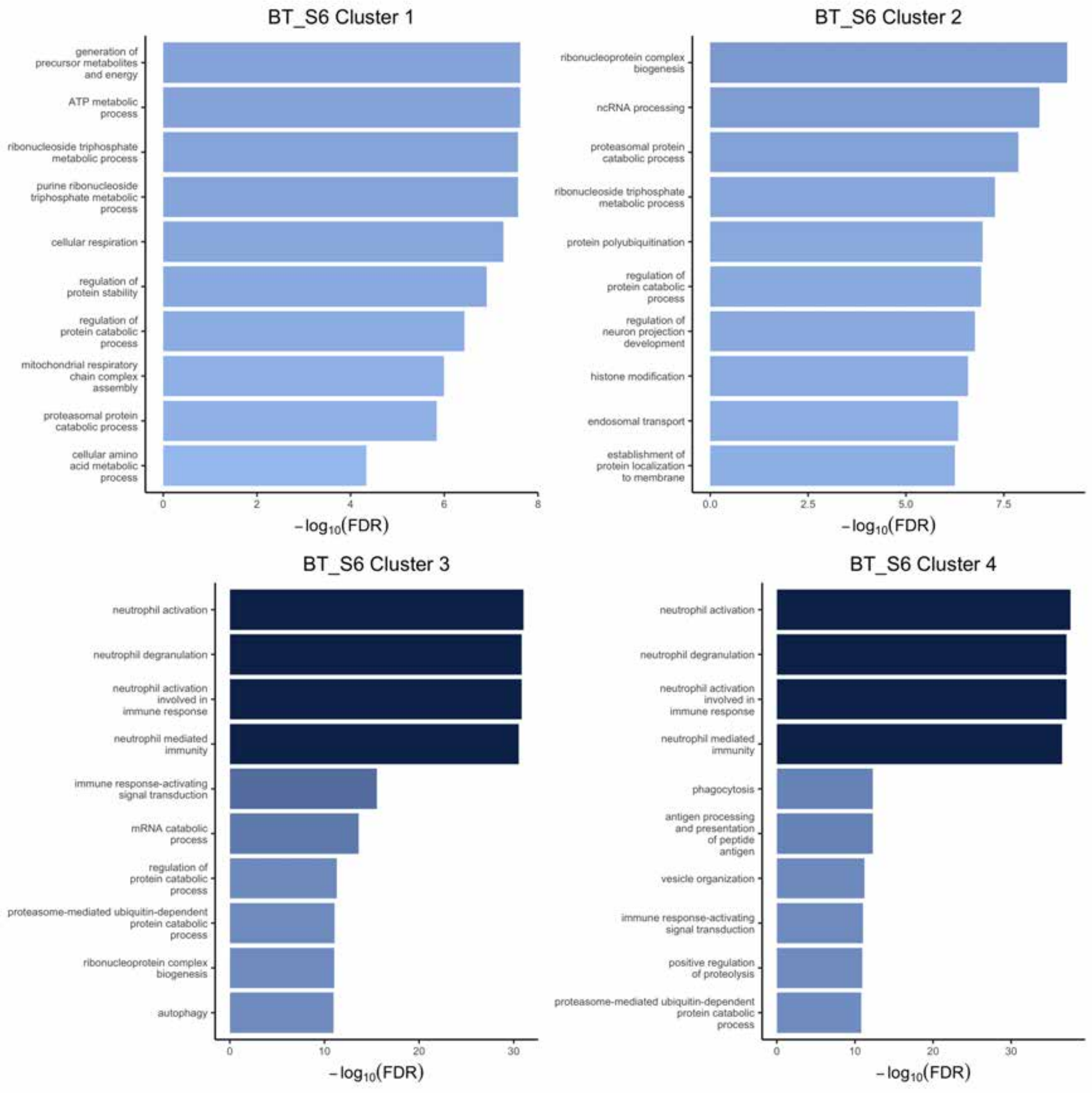

Supplementary Figure 15: Top 10 enriched GO-terms for the BT_S6 patient clusters.

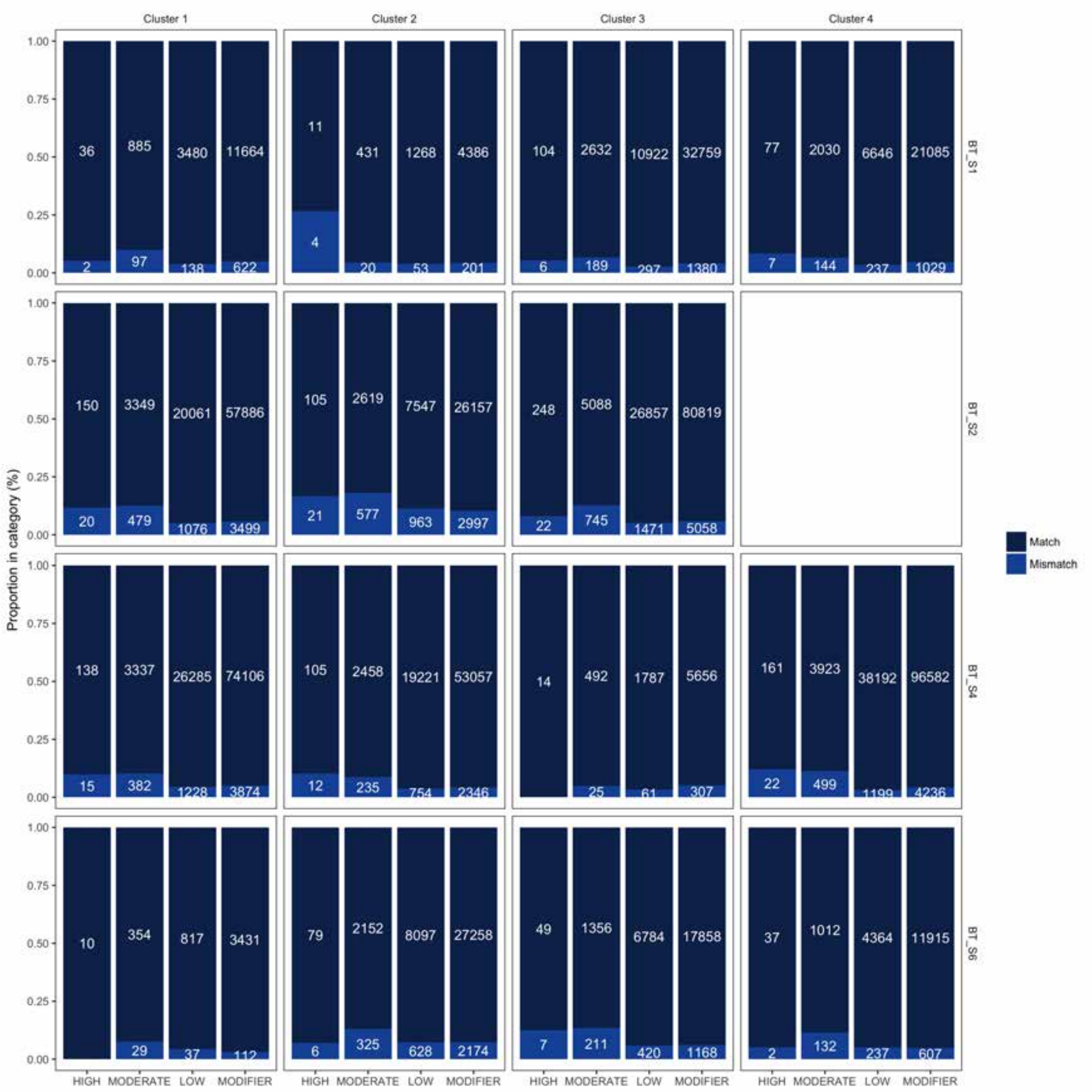

Supplementary Figure 16: Impact distribution across aggregate profiles for each GBM patient cluster.

Supplementary Table 5: Common and previously unknown missense variants across the three GBM neoplastic clusters in patients S1, S2 and S6.

Chromosome	Position	Gene	Impact	S1	S2	S6
6	29725552	HLA-F	MODERATE	G/G	G/G	A/G
12	46363770	SLC38A2	MODERATE	G/G	G/G	G/G
17	82938057	TBCD	MODERATE	G/G	G/G	G/G
