# Supplementary material for: Single-cell RNA-seq variant analysis for exploration of genetic heterogeneity in cancer
Source: Sci Rep. 2019 Jul 2;9:9524. doi: 10.1038/s41598-019-45934-1 (PMC6606766; doi:10.1038/s41598-019-45934-1)
Supplement: Supplementary file 2 — Supplementary code for data analysis [file 41598_2019_45934_MOESM2_ESM.pdf]

Single-cell RNA-seq variant analysis for exploration of genetic heterogeneity in cancer

Supplementary code 1

Erik Fasterius (1), Mathias Uhlén (1,2), Cristina Al-Khalili Szigyarto (1,2,*)

(1) School of Chemistry, Biotechnology and Health, KTH Royal Institute of Technology, Stockholm, Sweden
(2) Science for Life Laboratory, KTH Royal Institute of Technology, Solna, Sweden
(*) Corresponding author: caks@kth.se

Introduction

This document describes using the `varclust` python package for performing the following:

```
1) Creation of SNV profiles for each single cell
2) Pairwise comparisons between each profile
3) Clustering, grouping and visualisation of the different profiles
```

As the first step requires the several gigabytes' worth of VCF files (generated with the previously described RNA-seq variant calling pipeline) and the second comprises of several millions pairwise comparisons best performed in a massively parallell manner (such as on a computer cluster or a cloud service) only the last step is actually performed in this Jupyter notebook. The distance matrices are themselves also prohibitively large (~6.2 GB), which means that they cannot be supplied along with this publication, either. However, we will happily provide any of the files used for the analysis and all the raw data is publicly available for anyone to download from the GEO.

Creation and comparisons of SNV profiles

The `varclust` package can read VCF files and create SNV profiles for samples stored therein. It looks for variants passing whatever variant calling quality metrics defined by the variant calling method(s) itself, in addition to applying a hard filtering threshold of sequencing depth (default is 10). The functionality of `varclust` is described more in detail in its documentation. Here we utilise the command-line versions of the `varclust` modules that are automatically available upon installation of the package. Here we create all the various clustering figures shown in the article; most of the statistical analyses and their respective figures and tables are performed using R, the code of which is contained in its own `RMarkdown` document.

In [1]:

```python
# Figure formatting
from IPython.display import Image, HTML
CSS = """
.output {
    flex-direction: row;
}
"""
HTML('<style>{}</style>'.format(CSS))

# Show VarClust version used
import varclust
import pkg_resources
print(pkg_resources.get_distribution("varclust").version)
```

```
0.2.1
```

In [2]:
```
%%bash
# (1) Create full SNV profiles for each VCF in a directory
varclust_create_profiles vcf_dir profile_dir

# (2) Create a distance matrix for all the profiles in the directory
varclust_distance_matrix profile_dir distance_matrix.txt
```

Clustering of the GEO dataset

In [3]:
```
%%bash
# Cluster and visualise GEO distances (heatmap)
varclust_heatmap ../results/distances/geo/distances.geo.similarity_score.inner.
txt \
    ../results/figures/geo/heatmaps/geo_heatmap.png \
    --metadata-file ../metadata/metadata.geo.txt \
    --metadata-id GSE \
    --group-cols cell.line
```

In [4]:
```
%%bash
# Cluster and visualise GEO distances (tSNE)
varclust_tsne ../results/distances/geo/distances.geo.similarity_score.inner.txt
\
    ../results/figures/geo/tsnes/geo_tsne.png \
    --metadata-file ../metadata/metadata.geo.txt \
    --metadata-id GSE \
    --colour-col cell.line \
    --shape-col cell.line
```

In [5]:
```
x = Image("../results/figures/geo/heatmaps/geo_heatmap.png", width=450)
y = Image("../results/figures/geo/tsnes/geo_tsne.png", width=450)
display(x, y)
```

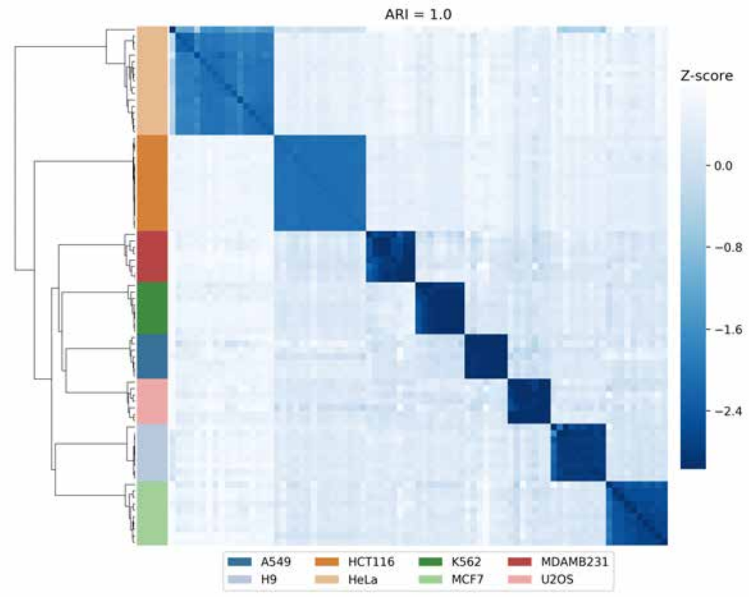

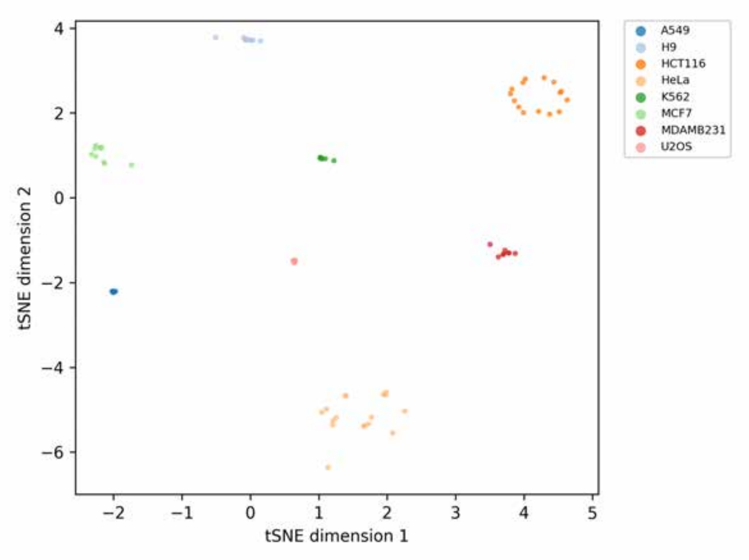

Clustering of the BC dataset

In [6]:
```
%%bash
# Cluster and visualise high quality cell BC distances (heatmap)
varclust_heatmap ../results/distances/bc/distances.bc.similarity_score.inner.HI
GH_MODERATE_LOW_MODIFIER.all.all.txt \
    ../results/figures/bc/heatmaps/bc_heatmap_all.png \
    --metadata-file ../metadata/metadata.bc.txt \
    --metadata-id SRR \
    --subset-groups "sample_quality,ok" \
    --group-cols patient
```

In [7]:
```
%%bash
# Cluster and visualise high quality cell  BC distances (tSNE)
varclust_tsne ../results/distances/bc/distances.bc.similarity_score.inner.HIGH_
MODERATE_LOW_MODIFIER.all.all.txt \
    ../results/figures/bc/tsnes/bc_tsne_all.png \
    --metadata-file ../metadata/metadata.bc.txt \
    --metadata-id SRR \
    --subset-groups "sample_quality,ok" \
    --colour-col patient \
    --shape-col patient
```

In [8]:
```
x = Image("../results/figures/bc/heatmaps/bc_heatmap_all.png", width=450)
y = Image("../results/figures/bc/tsnes/bc_tsne_all.png", width=450)
display(x, y)
```

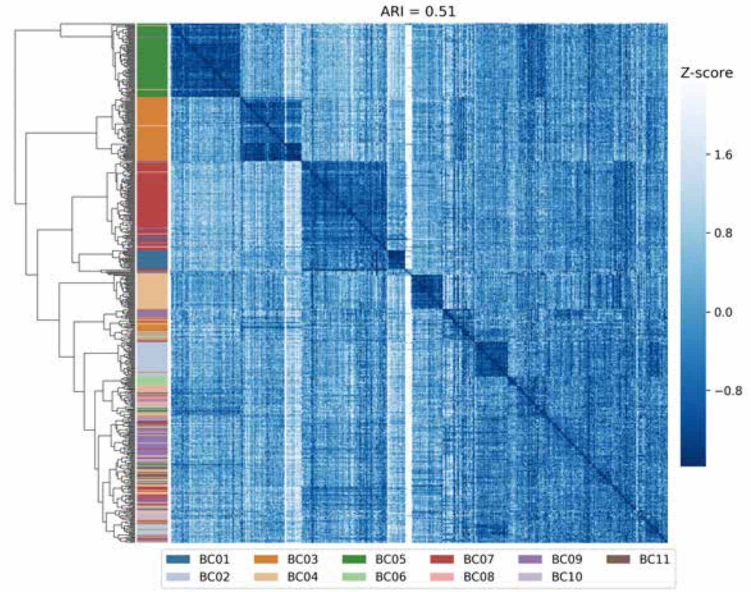

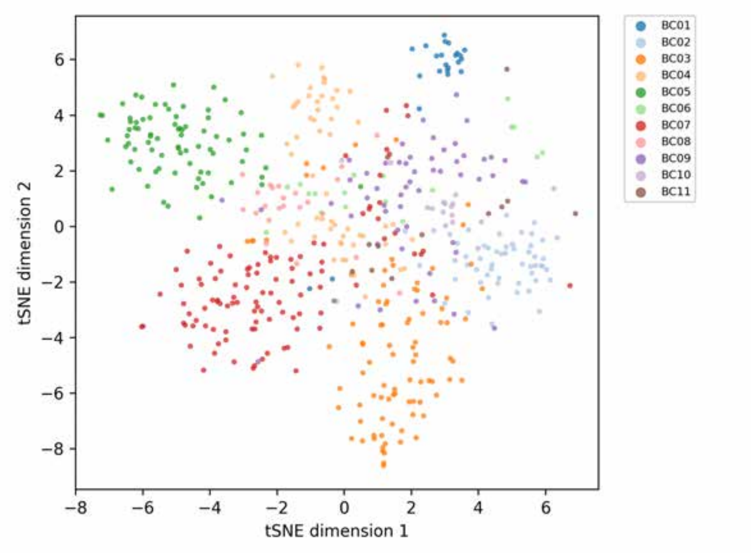

ARI-comparisons of clustering parameters in BC/GBM datasets

Comparisons of per-patient ARIs:

In [ ]:
```
%%bash

for DATASET in "bc" "gbm"; do

    # Initialise results file
    RESULTS=../results/clustering/patients/hac.results.$DATASET.txt
    printf "%s\t%s\t%s\n" "input" "cells" "ARI" > $RESULTS

    # Output directory
    OUTDIR=../results/clustering/patients/$DATASET

    # Distances
    for DISTANCE in ../results/distances/$DATASET/distances.$DATASET.similarity
_score.inner.*.txt; do

        # Distance name
        BASENAME=$(basename $DISTANCE)
        NAME=${BASENAME/.txt/}

        for SUBSET in 1 50 100 250 500 1000 2000 3000 4000 5000; do

            # Output file name
            OUTPUT=$OUTDIR/$NAME.$SUBSET.png
            echo "$NAME.$SUBSET"

            # Set subset
            if [ "$DATASET" == "bc" ]; then
                SUBSET_STRING="variants,>=$SUBSET;sample_quality,ok"
            else
                SUBSET_STRING="variants,>=$SUBSET"
            fi

            varclust_heatmap \
                "$DISTANCE" \
                "$OUTPUT" \
                -m ../metadata/metadata.$DATASET.txt \
                -M "SRR" \
                -l "ward" \
                -g "patient" \
                -A \
                -s $SUBSET_STRING \
                    >> $RESULTS
        done
    done
done
```

tSNE visualisations

In [10]:
```
%%bash

# tSNE visualisation (BC)
varclust_tsne \
    "../results/distances/bc/distances.bc.similarity_score.inner.HIGH_MODERATE_
LOW.None.all.txt" \
    "../results/figures/bc/tsnes/tsne.bc.HMLNoneAll.50.png" \
    -m "../metadata/metadata.bc.txt" -M "SRR" \
    -c "patient" \
    -s "patient" \
    -S "variants,>=50"
```

In [11]:

```
%%bash

# tSNE visualisation (GBM)
varclust_tsne \
    "../results/distances/gbm/distances.gbm.similarity_score.inner.HIGH_MODERAT
E_LOW.None.all.txt" \
    "../results/figures/gbm/tsnes/tsne.gbm.HMLNoneAll.50.png" \
    -m "../metadata/metadata.gbm.txt" -M "SRR" \
    -c "patient" \
    -s "patient" \
    -S "variants,>=50"
```

Intra-patient clustering of the GBM dataset

In [2]:

```
%%bash

OUTPUT_DIR="../results/clustering/clusters/gbm"
RESULTS="$OUTPUT_DIR/gbm_clusters.txt"
printf "%s\t%s\n" "SRR" "Cluster" \
    > $RESULTS

# Distance measure to use
DIST="../results/distances/gbm/distances.gbm.similarity_score.inner.HIGH_MODERA
TE_LOW.None.all.txt"

# For each GBM patient
export LC_ALL=C
for PATIENT in "BT_S1" "BT_S2" "BT_S4" "BT_S6"; do

    # Perform clustering
    varclust_heatmap \
        $DIST \
        "$OUTPUT_DIR/clusters.HMLNoneAll.1000.$PATIENT.png" \
        -c -C "$OUTPUT_DIR/clusters.$PATIENT.txt" \
        -m "../metadata/metadata.gbm.txt" -M "SRR" \
        -l "ward" \
        -g "patient" \
        -s "patient,$PATIENT;variants,>=1000"

    # tSNE visualisation
    varclust_tsne \
        $DIST \
        "$OUTPUT_DIR/tsne.HMLNoneAll.1000.$PATIENT.png" \
        -m "$OUTPUT_DIR/clusters.$PATIENT.txt" -M "id" \
        -c "cluster" \
        -s "cluster"

    # Add clusters to results file
    tail -n +2 "$OUTPUT_DIR/clusters.$PATIENT.txt" \
        >> $RESULTS
done
```

Aggregation of GBM profiles

In [ ]:

```
%%bash
#%%script false

# Cluster path
CLUSTERS="../results/clustering/clusters/gbm/"

# For each patient
for PATIENT in BT_S1 BT_S2 BT_S4 BT_S6; do

    # For each cluster
    for CLUSTER in $(tail -n +2 $CLUSTERS/clusters.$PATIENT.txt \
        | cut -f 2 | sort -u); do

        varclust_pseudo \
            "../data/gbm/profiles" \
            "seudo_${PATIENT}_${CLUSTER}.profile.txt" \
            -m "$CLUSTERS/clusters.$PATIENT.txt" \
            -M "id" \
            -s "cluster" \
            -S $CLUSTER
    done
done
```
