# Supplementary material for: Single-cell RNA-seq variant analysis for exploration of genetic heterogeneity in cancer
Source: Sci Rep. 2019 Jul 2;9:9524. doi: 10.1038/s41598-019-45934-1 (PMC6606766; doi:10.1038/s41598-019-45934-1)
Supplement: Supplementary file 3 — Supplementary code for generation of figures [file 41598_2019_45934_MOESM3_ESM.pdf]

Single-cell RNA-seq variant analysis for exploration of genetic heterogeneity in cancer

Supplementary code 2

Erik Fasterius (1), Mathias Uhlén (1,2), Cristina Al-Khalili Szigyarto (1,2,*) (1) School of Chemistry, Biotechnology and Health, KTH Royal Institute of Technology, Stockholm, Sweden
(2) Science for Life Laboratory, KTH Royal Institute of Technology, Solna, Sweden ()
Corresponding author: caks@kth.se

Packages

```
library("ggplot2")
library("scales")
library("dplyr")
library("tidyr")
library("seqCAT")
library("reshape2")
library("biomaRt")
library("clusterProfiler")
```

Common functions

```
# Create a function for reading, tidying and adding metadata
get_data <- function(dist_file, metadata, id_col, variant_subset) {

    # Read distance matrix
    dist <- read.table(dist_file, sep = "\t", header = TRUE,
                       stringsAsFactors = FALSE)
    names(dist) <- c("sample", names(dist)[2:ncol(dist)])

    # Read only lower triangle to remove symmetric entries
    dist[upper.tri(dist)] <- NA

    # Convert to long format
    data <- melt(dist)
    names(data) <- c("sample_1", "sample_2", "distance")
    data <- data[!is.na(data$distance), ]

    # Keep only relevant columns
    cols <- c(id_col, "patient", "cell.line", "location", "cell_class",
              "cancer_type", "sample_type", "sample_quality", "cell_type",
              "patient_and_location", "cluster", "variants")
    metadata <- unique(metadata[cols[cols %in% names(metadata)]])

    # Add metadata to tidy distance data
    data <- merge(data, metadata, by.x = "sample_1", by.y = id_col)
```

```
    data <- merge(data, metadata, by.x = "sample_2", by.y = id_col)
    names(data) <- gsub("\\.x", "_1", gsub("\\.y", "_2", names(data)))

    # Remove duplicated data
    data <- data[!duplicated(data[, c("sample_1", "sample_2")]), ]

    # Subset on variants
    data <- data[data$variants_1 >= variant_subset &
                 data$variants_2 >= variant_subset, ]

    # Return tidy data
    return(data)
}

# Function for plotting barplots with SEM
bar_sem <- function(data, x, output, width = 7, height = 7, facets = FALSE) {

    # Get x label
    x_label <- strsplit(x, "_")[[1]][1]
    x_label <- paste0(toupper(substr(x_label, 1, 1)),
                      tolower(substr(x_label, 2, nchar(x_label))))

    # Plot
    gg = ggplot(data, aes_string(x = x,
                                 y = "median_similarity")) +
        geom_bar(stat     = "identity",
                 position = position_dodge(),
                 fill     = "#1954a6") +
        geom_errorbar(aes(ymin = median_similarity - sem,
                          ymax = median_similarity + sem),
                      width = 0.3,
                      position = position_dodge()) +
        labs(x = x_label,
             y = "Median similarity score") +
        ylim(0, 1) +
        theme_bw() +
        theme(panel.grid.major = element_blank(),
              panel.grid.minor = element_blank(),
              strip.background = element_blank(),
              panel.border     = element_rect(colour = "black"))

    # Add facets (if applicable)
    if (facets) {
        gg <- gg + facet_grid(. ~ patient_1, scales = "free_x")
    }

    # Save to file
    ggsave(output, gg, width = width, height = height)
}
```

Analysis: ARI evaluation

Comparisons of ARIs for the different methods.

```
# Function for reading a plotting ARI data
plot_ari <- function(file, output) {

    # Read ARI data
    ari <- read.table(file, sep = "\t", header = TRUE,
                      stringsAsFactors = FALSE, quote="")

    # Separate distance information
    ari$input = gsub("inner.", "", ari$input)
    ari$input = gsub("distances.", "", ari$input)
    ari$input = gsub("bc.", "", ari$input)
    ari$input = gsub("gbm.", "", ari$input)
    ari <- ari %>%
        separate(input, into = c("metric", "impact", "class", "biotype",
                                 "subset"), sep = "\\.")

    # Factorise
    ari$impact <- gsub("_", "\n", ari$impact)
    ari$impact <- factor(ari$impact,
                         levels = c("HIGH\nMODERATE\nLOW\nMODIFIER",
                                    "HIGH\nMODERATE\nLOW",
                                    "HIGH\nMODERATE", "HIGH",
                                    "MODERATE", "LOW", "MODIFIER"))
    ari$subset <- factor(ari$subset, levels = c(1, 50, 100, 250, 500, 1000,
                                                2000, 3000, 4000, 5000))
    ari[ari$class == "all", "class"] <- "All"
    ari[ari$class == "None", "class"] <- "Unknown"
    ari[ari$biotype == "all", "biotype"] <- "All"
    ari[ari$biotype == "protein_coding", "biotype"] <- "Coding\nonly"

    for (distance in unique(ari$metric)) {

        current <- ari[ari$metric == distance, ]

        # Plot ARI data
        gg <- ggplot(current, aes(x    = biotype,
                                  y    = ARI,
                                  fill = class)) +
            geom_bar(stat     = "identity",
                     position = position_dodge()) +
            geom_text(aes(label = ARI),
                      vjust    = -0.3,
                      size     = 2,
                      position = position_dodge(0.9)) +
            geom_text(aes(label = cells),
                      vjust    = 1.3,
                      size     = 2,
                      colour   = "white",
                      position = position_dodge(0.9)) +
            facet_grid(subset ~ impact) +
```

```r
            theme_bw() +
            theme(legend.position = "bottom") +
            scale_fill_manual(values = c("#0d2d59", "#1954a6",
                                         "#4e8ce4", "#a6c6f2")) +
            ylim(0, 1.05) +
            labs(x    = "Biotype",
                 y    = "Adjusted Rand Index",
                 fill = "Variant class")

        # Save plot to file
        output_final <- paste0(output, distance, ".png")
        ggsave(output_final, gg, dpi = 300, height = 9, width = 9)
    }
}
plot_ari("../results/clustering/hac.results.bc.txt",
         "../results/figures/ARI.bc.")
plot_ari("../results/clustering/hac.results.gbm.txt",
         "../results/figures/ARI.gbm.")
```

Analysis: breast cancer

BC: heterogeneity across patients and locations

```
# Read BC data
metadata_bc <- read.table("../metadata/metadata.bc.txt", sep = "\t",
                          header = TRUE, quote = "", stringsAsFactors = FALSE,
                          fill = TRUE)

# Loop over subsets
subsets <- c("HIGH_MODERATE_LOW.None.all",
             "HIGH.all.all",
             "HIGH.None.all",
             "MODERATE.all.all",
             "MODERATE.None.all",
             "LOW.all.all",
             "LOW.None.all",
             "MODIFIER.all.all",
             "MODIFIER.None.all")

for (subset in subsets) {

    # Progress
    print(paste0("-----------------------", subset, "-----------------------"))

    # Read data
    dist <- paste0("../results/distances/bc/distances.bc.similarity_score.",
                   "inner.", subset, ".txt")
    bc <- get_data(dist, metadata_bc, "SRR", 50)
    bc <- bc[bc$sample_quality_1 == "ok" & bc$sample_quality_2 == "ok", ]

    # Filter data
    bc_filtered <- bc %>%
        filter(patient_and_location_1 == patient_and_location_2) %>%
        filter(sample_1 != sample_2) %>%
        filter(sample_type_1 == "individual_cell" &
               sample_type_2 == "individual_cell")

    # Per-patient-and-location heterogeneity and significance testing
    bc_patients_and_locations <- bc_filtered %>%
        group_by(patient_and_location_1) %>%
        summarise(median_similarity = 1 - round(median(distance,
                                                       na.rm = TRUE), 3),
                  count = n(),
                  sd    = sd(1 - distance),
                  cells = as.integer(1/2 + sqrt(1/4 + 2 * n()))) %>%
        mutate(sem = sd / sqrt(count))
    print(bc_patients_and_locations)
    output <- paste0("../results/figures/heterogeneity.bc.patients_and_loc.",
                     subset, ".png")
    bar_sem(bc_patients_and_locations, "patient_and_location_1", output,
            width = 9, height = 7)
```

```
    print(bc_filtered %>%
        filter(patient_1 == "BC03" | patient_1 == "BC07") %>%
        group_by(patient_1, location_1) %>%
        summarise(similarity = list(1 - distance)) %>%
        spread(location_1, similarity) %>%
        group_by(patient_1) %>%
        mutate(p_value = t.test(unlist(core_tumour),
                                unlist(lymph_node))$p.value))

    # Per-subtype heterogeneity and ANOVA
    bc_subtypes <- bc_filtered %>%
        filter(location_1 == "core_tumour" & location_2 == "core_tumour") %>%
        filter(cancer_type_1 == cancer_type_2)

    bc_subtypes_summary <- bc_subtypes %>%
        group_by(cancer_type_1) %>%
        summarise(median_similarity = 1 - round(median(distance,
                                                         na.rm = TRUE), 3),
                  count = n(),
                  sd    = sd(1 - distance),
                  cells = as.integer(1/2 + sqrt(1/4 + 2 * n()))) %>%
        mutate(sem = sd / sqrt(count))
    print(bc_subtypes_summary)
    output <- paste0("../results/figures/heterogeneity.bc.cancer_types.",
                     subset, ".png")
    bar_sem(bc_subtypes_summary, "cancer_type_1", output)
    anova <- aov(lm(1 - distance ~ cancer_type_1, data = bc_subtypes))
    summary(anova)
    tukey <- TukeyHSD(anova)
    print(tukey)
}
```

```
## [1] "-----------------------HIGH_MODERATE_LOW.None.all-----------------------"

## Using sample as id variables

## # A tibble: 13 x 6
##    patient_and_location_1 median_similarity count     sd cells     sem
##    <chr>                              <dbl> <int>  <dbl> <int>   <dbl>
##  1 BC01                               0.615   231 0.112     22 0.00737
##  2 BC02                               0.704  1378 0.0621    53 0.00167
##  3 BC03                               0.7     528 0.0745    33 0.00324
##  4 BC03LN                             0.714  1378 0.0897    53 0.00242
##  5 BC04                               0.698  1485 0.0739    55 0.00192
##  6 BC05                               0.682  2775 0.0806    75 0.00153
##  7 BC06                               0.75    153 0.0691    18 0.00559
##  8 BC07                               0.714  1225 0.0632    50 0.00181
##  9 BC07LN                             0.75   1326 0.0772    52 0.00212
## 10 BC08                               0.688   231 0.0589    22 0.00388
## 11 BC09                               0.643  1485 0.112     55 0.00291
## 12 BC10                               0.643   105 0.0633    15 0.00618
## 13 BC11                               0.682    55 0.0793    11 0.0107

## # A tibble: 2 x 4
## # Groups:   patient_1 [2]
```

```
##   patient_1 core_tumour   lymph_node     p_value
##   <chr>     <list>        <list>           <dbl>
## 1 BC03      <dbl [528]>   <dbl [1,378]> 2.06e- 6
## 2 BC07      <dbl [1,225]> <dbl [1,326]> 1.89e-29
## # A tibble: 4 x 6
##   cancer_type_1 median_similarity count     sd cells     sem
##   <chr>                     <dbl> <int>  <dbl> <int>   <dbl>
## 1 ER+                       0.696  1609 0.0793    57 0.00198
## 2 ER+/HER2+                 0.7     528 0.0745    33 0.00324
## 3 HER2+                     0.688  4413 0.0795    94 0.00120
## 4 TNBC                      0.684  3101 0.0972    79 0.00174

##   Tukey multiple comparisons of means
##     95% family-wise confidence level
##
## Fit: aov(formula = lm(1 - distance ~ cancer_type_1, data = bc_subtypes))
##
## $cancer_type_1
##                         diff          lwr           upr     p adj
## ER+/HER2+-ER+    0.003037271 -0.007956609  0.0140311511 0.8932303
## HER2+-ER+       -0.007985672 -0.014369323 -0.0016020217 0.0071791
## TNBC-ER+        -0.015528293 -0.022263101 -0.0087934840 0.0000000
## HER2+-ER+/HER2+ -0.011022943 -0.021117034 -0.0009288524 0.0258776
## TNBC-ER+/HER2+  -0.018565564 -0.028885318 -0.0082458096 0.0000228
## TNBC-HER2+      -0.007542620 -0.012679051 -0.0024061894 0.0009322
##
## [1] "-----------------------HIGH.all.all-----------------------"

## Using sample as id variables

## # A tibble: 13 x 6
##    patient_and_location_1 median_similarity count     sd cells     sem
##    <chr>                              <dbl> <int>  <dbl> <int>   <dbl>
##  1 BC01                               0.3     231 0.139     22 0.00917
##  2 BC02                               0.286  1378 0.0919    53 0.00248
##  3 BC03                               0.375   528 0.131     33 0.00568
##  4 BC03LN                             0.444  1378 0.132     53 0.00356
##  5 BC04                               0.375  1485 0.100     55 0.00260
##  6 BC05                               0.5    2775 0.0882    75 0.00167
##  7 BC06                               0.4     153 0.0904    18 0.00731
##  8 BC07                               0.286  1225 0.107     50 0.00307
##  9 BC07LN                             0.375  1326 0.101     52 0.00277
## 10 BC08                               0.167   231 0.0696    22 0.00458
## 11 BC09                               0.375  1485 0.108     55 0.00279
## 12 BC10                               0.286   105 0.0975    15 0.00952
## 13 BC11                               0.4      55 0.0881    11 0.0119

## # A tibble: 2 x 4
## # Groups:   patient_1 [2]
##   patient_1 core_tumour   lymph_node     p_value
##   <chr>     <list>        <list>           <dbl>
## 1 BC03      <dbl [528]>   <dbl [1,378]> 3.19e-22
## 2 BC07      <dbl [1,225]> <dbl [1,326]> 9.21e-40
## # A tibble: 4 x 6
##   cancer_type_1 median_similarity count    sd cells     sem
##   <chr>                     <dbl> <int> <dbl> <int>   <dbl>
```

```
## 1 ER+                       0.286  1609 0.102    57 0.00254
## 2 ER+/HER2+                 0.375   528 0.131    33 0.00568
## 3 HER2+                     0.455  4413 0.103    94 0.00155
## 4 TNBC                      0.333  3101 0.113    79 0.00202

##   Tukey multiple comparisons of means
##     95% family-wise confidence level
##
## Fit: aov(formula = lm(1 - distance ~ cancer_type_1, data = bc_subtypes))
##
## $cancer_type_1
##                       diff         lwr         upr p adj
## ER+/HER2+-ER+    0.08164085  0.06777359  0.09550810     0
## HER2+-ER+        0.17412315  0.16607106  0.18217524     0
## TNBC-ER+         0.04540743  0.03691240  0.05390246     0
## HER2+-ER+/HER2+  0.09248230  0.07975001  0.10521460     0
## TNBC-ER+/HER2+  -0.03623342 -0.04925036 -0.02321647     0
## TNBC-HER2+      -0.12871572 -0.13519462 -0.12223682     0
##
## [1] "----------------------HIGH.None.all----------------------"

## Using sample as id variables

## # A tibble: 13 x 6
##    patient_and_location_1 median_similarity count     sd cells      sem
##    <chr>                              <dbl> <int>  <dbl> <int>    <dbl>
##  1 BC01                               0.167   231 0.0428    22 0.00282
##  2 BC02                               0.167  1378 0.0639    53 0.00172
##  3 BC03                               0.167   528 0.0640    33 0.00278
##  4 BC03LN                             0.286  1378 0.0657    53 0.00177
##  5 BC04                               0.286  1485 0.0578    55 0.00150
##  6 BC05                               0.167  2775 0.0514    75 0.000977
##  7 BC06                               0.286   153 0.0779    18 0.00630
##  8 BC07                               0.167  1225 0.0674    50 0.00192
##  9 BC07LN                             0.286  1326 0.0727    52 0.00200
## 10 BC08                               0.167   231 0.0389    22 0.00256
## 11 BC09                               0.25   1485 0.0809    55 0.00210
## 12 BC10                               0.167   105 0.0554    15 0.00541
## 13 BC11                               0.167    55 0.0603    11 0.00814

## # A tibble: 2 x 4
## # Groups:   patient_1 [2]
##   patient_1 core_tumour   lymph_node     p_value
##   <chr>     <list>        <list>           <dbl>
## 1 BC03      <dbl [528]>   <dbl [1,378]> 1.96e-71
## 2 BC07      <dbl [1,225]> <dbl [1,326]> 1.17e-47
## # A tibble: 4 x 6
##   cancer_type_1 median_similarity count     sd cells     sem
##   <chr>                     <dbl> <int>  <dbl> <int>   <dbl>
## 1 ER+                       0.167  1609 0.0620    57 0.00155
## 2 ER+/HER2+                 0.167   528 0.0640    33 0.00278
## 3 HER2+                     0.167  4413 0.0808    94 0.00122
## 4 TNBC                      0.167  3101 0.0745    79 0.00134

##   Tukey multiple comparisons of means
##     95% family-wise confidence level
```

```
##
## Fit: aov(formula = lm(1 - distance ~ cancer_type_1, data = bc_subtypes))
##
## $cancer_type_1
##                         diff           lwr          upr     p adj
## ER+/HER2+-ER+    0.007704705 -0.0019684970  0.017377907 0.1711048
## HER2+-ER+        0.031066620  0.0254498277  0.036683412 0.0000000
## TNBC-ER+         0.016557034  0.0106312680  0.022482801 0.0000000
## HER2+-ER+/HER2+  0.023361915  0.0144804117  0.032243418 0.0000000
## TNBC-ER+/HER2+   0.008852329 -0.0002277286  0.017932387 0.0591840
## TNBC-HER2+      -0.014509586 -0.0190289852 -0.009990186 0.0000000
##
## [1] "-----------------------MODERATE.all.all-----------------------"

## Using sample as id variables

## # A tibble: 13 x 6
##    patient_and_location_1 median_similarity count     sd cells      sem
##    <chr>                              <dbl> <int>  <dbl> <int>    <dbl>
##  1 BC01                               0.836   231 0.0646    22 0.00425
##  2 BC02                               0.821  1378 0.0494    53 0.00133
##  3 BC03                               0.792   528 0.0549    33 0.00239
##  4 BC03LN                             0.806  1378 0.0564    53 0.00152
##  5 BC04                               0.804  1485 0.0515    55 0.00134
##  6 BC05                               0.845  2775 0.0463    75 0.000879
##  7 BC06                               0.812   153 0.0519    18 0.00420
##  8 BC07                               0.797  1225 0.0531    50 0.00152
##  9 BC07LN                             0.809  1326 0.0511    52 0.00140
## 10 BC08                               0.76    231 0.0778    22 0.00512
## 11 BC09                               0.779  1485 0.0695    55 0.00180
## 12 BC10                               0.812   105 0.0760    15 0.00742
## 13 BC11                               0.849    55 0.0439    11 0.00591

## # A tibble: 2 x 4
## # Groups:   patient_1 [2]
##   patient_1 core_tumour   lymph_node          p_value
##   <chr>     <list>        <list>                <dbl>
## 1 BC03      <dbl [528]>   <dbl [1,378]> 0.00480
## 2 BC07      <dbl [1,225]> <dbl [1,326]> 0.00000000151
## # A tibble: 4 x 6
##   cancer_type_1 median_similarity count     sd cells      sem
##   <chr>                     <dbl> <int>  <dbl> <int>    <dbl>
## 1 ER+                       0.824  1609 0.0518    57 0.00129
## 2 ER+/HER2+                 0.792   528 0.0549    33 0.00239
## 3 HER2+                     0.831  4413 0.0518    94 0.000780
## 4 TNBC                      0.789  3101 0.0662    79 0.00119

##   Tukey multiple comparisons of means
##     95% family-wise confidence level
##
## Fit: aov(formula = lm(1 - distance ~ cancer_type_1, data = bc_subtypes))
##
## $cancer_type_1
##                         diff          lwr          upr     p adj
## ER+/HER2+-ER+   -0.025852271 -0.033195829 -0.018508712 0.0000000
## HER2+-ER+        0.007811570  0.003547497  0.012075644 0.0000151
```

```
## TNBC-ER+        -0.035455179 -0.039953814 -0.030956543 0.0000000
## HER2+-ER+/HER2+  0.033663841  0.026921313  0.040406370 0.0000000
## TNBC-ER+/HER2+  -0.009602908 -0.016496172 -0.002709644 0.0019621
## TNBC-HER2+      -0.043266749 -0.046697720 -0.039835778 0.0000000
##
## [1] "-----------------------MODERATE.None.all-----------------------"

## Using sample as id variables

## # A tibble: 13 x 6
##    patient_and_location_1 median_similarity count     sd cells     sem
##    <chr>                              <dbl> <int>  <dbl> <int>   <dbl>
##  1 BC01                               0.444   231 0.144     22 0.00946
##  2 BC02                               0.444  1378 0.104     53 0.00281
##  3 BC03                               0.467   528 0.102     33 0.00444
##  4 BC03LN                             0.545  1378 0.127     53 0.00341
##  5 BC04                               0.5    1485 0.132     55 0.00343
##  6 BC05                               0.5    2775 0.0948    75 0.00180
##  7 BC06                               0.444   153 0.148     18 0.0120
##  8 BC07                               0.462  1225 0.126     50 0.00361
##  9 BC07LN                             0.562  1326 0.134     52 0.00368
## 10 BC08                               0.444   231 0.0913    22 0.00601
## 11 BC09                               0.444  1485 0.152     55 0.00395
## 12 BC10                               0.444   105 0.0747    15 0.00729
## 13 BC11                               0.5      55 0.135     11 0.0182

## # A tibble: 2 x 4
## # Groups:   patient_1 [2]
##   patient_1 core_tumour   lymph_node     p_value
##   <chr>     <list>        <list>           <dbl>
## 1 BC03      <dbl [528]>   <dbl [1,378]> 6.37e-42
## 2 BC07      <dbl [1,225]> <dbl [1,326]> 5.69e-44
## # A tibble: 4 x 6
##   cancer_type_1 median_similarity count    sd cells     sem
##   <chr>                     <dbl> <int> <dbl> <int>   <dbl>
## 1 ER+                       0.444  1609 0.111    57 0.00277
## 2 ER+/HER2+                 0.467   528 0.102    33 0.00444
## 3 HER2+                     0.5    4413 0.111    94 0.00167
## 4 TNBC                      0.455  3101 0.137    79 0.00246

##   Tukey multiple comparisons of means
##     95% family-wise confidence level
##
## Fit: aov(formula = lm(1 - distance ~ cancer_type_1, data = bc_subtypes))
##
## $cancer_type_1
##                         diff          lwr          upr     p adj
## ER+/HER2+-ER+    0.052094295  0.036696883  0.067491708 0.0000000
## HER2+-ER+        0.060481465  0.051540881  0.069422048 0.0000000
## TNBC-ER+         0.027745844  0.018313448  0.037178239 0.0000000
## HER2+-ER+/HER2+  0.008387169 -0.005750049  0.022524387 0.4228182
## TNBC-ER+/HER2+  -0.024348452 -0.038801721 -0.009895182 0.0000893
## TNBC-HER2+      -0.032735621 -0.039929418 -0.025541824 0.0000000
##
## [1] "-----------------------LOW.all.all-----------------------"
```

```
## Using sample as id variables

## # A tibble: 13 x 6
##    patient_and_location_1 median_similarity count     sd cells      sem
##    <chr>                              <dbl> <int>  <dbl> <int>    <dbl>
##  1 BC01                               0.881   231 0.0249    22 0.00164
##  2 BC02                               0.887  1378 0.0306    53 0.000824
##  3 BC03                               0.861   528 0.0397    33 0.00173
##  4 BC03LN                             0.853  1378 0.0395    53 0.00106
##  5 BC04                               0.884  1485 0.0296    55 0.000768
##  6 BC05                               0.884  2775 0.0255    75 0.000483
##  7 BC06                               0.867   153 0.0428    18 0.00346
##  8 BC07                               0.872  1225 0.0283    50 0.000809
##  9 BC07LN                             0.875  1326 0.0293    52 0.000804
## 10 BC08                               0.852   231 0.0594    22 0.00391
## 11 BC09                               0.847  1485 0.0432    55 0.00112
## 12 BC10                               0.896   105 0.0394    15 0.00385
## 13 BC11                               0.89     55 0.0211    11 0.00285

## # A tibble: 2 x 4
## # Groups:   patient_1 [2]
##   patient_1 core_tumour   lymph_node          p_value
##   <chr>     <list>        <list>                <dbl>
## 1 BC03      <dbl [528]>   <dbl [1,378]> 0.00000000739
## 2 BC07      <dbl [1,225]> <dbl [1,326]> 0.00318
## # A tibble: 4 x 6
##   cancer_type_1 median_similarity count     sd cells      sem
##   <chr>                     <dbl> <int>  <dbl> <int>    <dbl>
## 1 ER+                       0.885  1609 0.0298    57 0.000744
## 2 ER+/HER2+                 0.861   528 0.0397    33 0.00173
## 3 HER2+                     0.883  4413 0.0281    94 0.000423
## 4 TNBC                      0.862  3101 0.0423    79 0.000760

##   Tukey multiple comparisons of means
##     95% family-wise confidence level
##
## Fit: aov(formula = lm(1 - distance ~ cancer_type_1, data = bc_subtypes))
##
## $cancer_type_1
##                         diff          lwr           upr     p adj
## ER+/HER2+-ER+   -0.025422861 -0.029833088 -0.0210126333 0.0000000
## HER2+-ER+       -0.002489071 -0.005049891  0.0000717493 0.0603462
## TNBC-ER+        -0.027344276 -0.030045965 -0.0246425882 0.0000000
## HER2+-ER+/HER2+  0.022933790  0.018884515  0.0269830640 0.0000000
## TNBC-ER+/HER2+  -0.001921416 -0.006061216  0.0022183839 0.6315585
## TNBC-HER2+      -0.024855206 -0.026915700 -0.0227947112 0.0000000
##
## [1] "----------------------LOW.None.all----------------------"

## Using sample as id variables

## # A tibble: 13 x 6
##    patient_and_location_1 median_similarity count     sd cells     sem
##    <chr>                              <dbl> <int>  <dbl> <int>   <dbl>
##  1 BC01                               0.583   231 0.105     22 0.00690
##  2 BC02                               0.667  1378 0.0611    53 0.00165
```

```
##  3 BC03                                0.647   528 0.0828    33 0.00360
##  4 BC03LN                              0.667  1378 0.0956    53 0.00258
##  5 BC04                                0.625  1485 0.0788    55 0.00205
##  6 BC05                                0.611  2775 0.0931    75 0.00177
##  7 BC06                                0.714   153 0.0653    18 0.00528
##  8 BC07                                0.688  1225 0.0596    50 0.00170
##  9 BC07LN                              0.714  1326 0.0701    52 0.00192
## 10 BC08                                0.647   231 0.0588    22 0.00387
## 11 BC09                                0.615  1485 0.0918    55 0.00238
## 12 BC10                                0.571   105 0.0730    15 0.00713
## 13 BC11                                0.667    55 0.0702    11 0.00946

## # A tibble: 2 x 4
## # Groups:   patient_1 [2]
##   patient_1 core_tumour   lymph_node     p_value
##   <chr>     <list>        <list>           <dbl>
## 1 BC03      <dbl [528]>   <dbl [1,378]> 1.14e- 3
## 2 BC07      <dbl [1,225]> <dbl [1,326]> 5.14e-16
## # A tibble: 4 x 6
##   cancer_type_1 median_similarity count     sd cells     sem
##   <chr>                     <dbl> <int>  <dbl> <int>   <dbl>
## 1 ER+                       0.667  1609 0.0789    57 0.00197
## 2 ER+/HER2+                 0.647   528 0.0828    33 0.00360
## 3 HER2+                     0.621  4413 0.0904    94 0.00136
## 4 TNBC                      0.65   3101 0.0870    79 0.00156

##   Tukey multiple comparisons of means
##     95% family-wise confidence level
##
## Fit: aov(formula = lm(1 - distance ~ cancer_type_1, data = bc_subtypes))
##
## $cancer_type_1
##                          diff          lwr          upr     p adj
## ER+/HER2+-ER+   -0.013723521 -0.024944940 -0.002502102 0.0091154
## HER2+-ER+       -0.039559749 -0.046075521 -0.033043976 0.0000000
## TNBC-ER+        -0.011608445 -0.018482643 -0.004734246 0.0000852
## HER2+-ER+/HER2+ -0.025836228 -0.036139235 -0.015533220 0.0000000
## TNBC-ER+/HER2+   0.002115076 -0.008418265  0.012648417 0.9553056
## TNBC-HER2+       0.027951304  0.022708565  0.033194043 0.0000000
##
## [1] "----------------------MODIFIER.all.all----------------------"

## Using sample as id variables

## # A tibble: 13 x 6
##    patient_and_location_1 median_similarity count     sd cells      sem
##    <chr>                              <dbl> <int>  <dbl> <int>    <dbl>
##  1 BC01                               0.881   231 0.0222    22 0.00146
##  2 BC02                               0.908  1378 0.0234    53 0.000631
##  3 BC03                               0.877   528 0.0342    33 0.00149
##  4 BC03LN                             0.866  1378 0.0369    53 0.000995
##  5 BC04                               0.89   1485 0.0249    55 0.000647
##  6 BC05                               0.891  2775 0.0184    75 0.000349
##  7 BC06                               0.884   153 0.0251    18 0.00203
##  8 BC07                               0.892  1225 0.0272    50 0.000778
##  9 BC07LN                             0.893  1326 0.0243    52 0.000666
```

```
## 10 BC08                          0.923   231 0.0249    22 0.00164
## 11 BC09                          0.899  1485 0.0279    55 0.000723
## 12 BC10                          0.921   105 0.0330    15 0.00322
## 13 BC11                          0.902    55 0.0261    11 0.00351

## # A tibble: 2 x 4
## # Groups:   patient_1 [2]
##   patient_1 core_tumour   lymph_node     p_value
##   <chr>     <list>        <list>           <dbl>
## 1 BC03      <dbl [528]>   <dbl [1,378]> 4.08e-10
## 2 BC07      <dbl [1,225]> <dbl [1,326]> 1.41e- 1
## # A tibble: 4 x 6
##   cancer_type_1 median_similarity count     sd cells      sem
##   <chr>                     <dbl> <int>  <dbl> <int>    <dbl>
## 1 ER+                       0.905  1609 0.0248    57 0.000618
## 2 ER+/HER2+                 0.877   528 0.0342    33 0.00149
## 3 HER2+                     0.891  4413 0.0211    94 0.000318
## 4 TNBC                      0.898  3101 0.0290    79 0.000521

##   Tukey multiple comparisons of means
##     95% family-wise confidence level
##
## Fit: aov(formula = lm(1 - distance ~ cancer_type_1, data = bc_subtypes))
##
## $cancer_type_1
##                         diff          lwr          upr p adj
## ER+/HER2+-ER+   -0.030407009 -0.033669808 -0.027144211     0
## HER2+-ER+       -0.013978936 -0.015873496 -0.012084376     0
## TNBC-ER+        -0.006257545 -0.008256323 -0.004258767     0
## HER2+-ER+/HER2+  0.016428073  0.013432317  0.019423830     0
## TNBC-ER+/HER2+   0.024149464  0.021086735  0.027212194     0
## TNBC-HER2+       0.007721391  0.006196984  0.009245797     0
##
## [1] "----------------------MODIFIER.None.all----------------------"

## Using sample as id variables

## # A tibble: 13 x 6
##    patient_and_location_1 median_similarity count     sd cells     sem
##    <chr>                              <dbl> <int>  <dbl> <int>   <dbl>
##  1 BC01                               0.739   231 0.133     22 0.00875
##  2 BC02                               0.788  1378 0.136     53 0.00367
##  3 BC03                               0.814   528 0.111     33 0.00484
##  4 BC03LN                             0.821  1378 0.0896    53 0.00242
##  5 BC04                               0.84   1485 0.0740    55 0.00192
##  6 BC05                               0.861  2775 0.0648    75 0.00123
##  7 BC06                               0.828   153 0.0915    18 0.00740
##  8 BC07                               0.833  1225 0.102     50 0.00291
##  9 BC07LN                             0.846  1326 0.0833    52 0.00229
## 10 BC08                               0.865   231 0.0757    22 0.00498
## 11 BC09                               0.783  1485 0.110     55 0.00285
## 12 BC10                               0.852   105 0.110     15 0.0108
## 13 BC11                               0.822    55 0.0733    11 0.00988

## # A tibble: 2 x 4
## # Groups:   patient_1 [2]
```

```
##   patient_1 core_tumour   lymph_node        p_value
##   <chr>     <list>        <list>              <dbl>
## 1 BC03      <dbl [528]>   <dbl [1,378]> 0.000417
## 2 BC07      <dbl [1,225]> <dbl [1,326]> 0.000000964
## # A tibble: 4 x 6
##   cancer_type_1 median_similarity count     sd cells     sem
##   <chr>                     <dbl> <int>  <dbl> <int>   <dbl>
## 1 ER+                       0.779  1609 0.137     57 0.00342
## 2 ER+/HER2+                 0.814   528 0.111     33 0.00484
## 3 HER2+                     0.853  4413 0.0699    94 0.00105
## 4 TNBC                      0.811  3101 0.108     79 0.00193

##   Tukey multiple comparisons of means
##     95% family-wise confidence level
##
## Fit: aov(formula = lm(1 - distance ~ cancer_type_1, data = bc_subtypes))
##
## $cancer_type_1
##                         diff          lwr         upr     p adj
## ER+/HER2+-ER+    0.026865427  0.014117456  0.03961340 0.0000004
## HER2+-ER+        0.084736013  0.077333841  0.09213819 0.0000000
## TNBC-ER+         0.032190296  0.024380938  0.03999965 0.0000000
## HER2+-ER+/HER2+  0.057870586  0.046165966  0.06957521 0.0000000
## TNBC-ER+/HER2+   0.005324869 -0.006641419  0.01729116 0.6625906
## TNBC-HER2+      -0.052545717 -0.058501674 -0.04658976 0.0000000
```

Analysis: glioblastoma

GBM: heterogeneity

```
# Read GBM data
dist = paste0("../results/distances/gbm/distances.gbm.similarity_score.inner.",
              "HIGH_MODERATE_LOW_MODIFIER.None.all.txt")
metadata_gbm <- read.table("../metadata/metadata.gbm.txt", sep = "\t",
                           header = TRUE, quote = "", stringsAsFactors = FALSE,
                           fill = TRUE)
gbm = get_data(dist, metadata_gbm, "SRR", 1000)
```

```
## Using sample as id variables
```

```
# Filter data
gbm_filtered = gbm %>%
    filter(patient_1 == patient_2) %>%
    filter(sample_1 != sample_2)

# Per-patient heterogeneity
gbm_patients <- gbm_filtered %>%
    group_by(patient_1) %>%
    summarise(median_similarity = 1 - round(median(distance, na.rm = TRUE), 3),
              count = n(),
              sd    = sd(1 - distance),
              cells = as.integer(1/2 + sqrt(1/4 + 2 * n()))) %>%
    mutate(sem = sd / sqrt(count))
gbm_patients
```

```
## # A tibble: 4 x 6
##   patient_1 median_similarity  count     sd cells       sem
##   <chr>                 <dbl>  <int>  <dbl> <int>     <dbl>
## 1 BT_S1                 0.5    108811 0.175    467 0.000532
## 2 BT_S2                 0.828 578350 0.0669   1076 0.0000880
## 3 BT_S4                 0.654 866586 0.159    1317 0.000171
## 4 BT_S6                 0.667  57291 0.147     339 0.000615
```

```
bar_sem(gbm_patients, "patient_1",
        "../results/figures/heterogeneity.gbm.patients.png")

# Per-patient-and-cluster heterogeneity
gbm_patients_and_clusters <- gbm_filtered %>%
    filter(cluster_1 == cluster_2) %>%
    group_by(patient_1, cluster_1) %>%
    summarise(median_similarity = 1 - round(median(distance, na.rm = TRUE), 3),
              count = n(),
              sd    = sd(1 - distance),
              cells = as.integer(1/2 + sqrt(1/4 + 2 * n()))) %>%
    mutate(sem = sd / sqrt(count))
gbm_patients_and_clusters
```

```
## # A tibble: 15 x 7
## # Groups:   patient_1 [4]
##    patient_1 cluster_1 median_similarity  count     sd cells      sem
##    <chr>     <chr>                 <dbl>  <int>  <dbl> <int>    <dbl>
```

```
##   1 BT_S1     cluster_1             0.767   1485 0.118     55 0.00307
##   2 BT_S1     cluster_2             0.647   1035 0.181     46 0.00563
##   3 BT_S1     cluster_3             0.625    496 0.107     32 0.00481
##   4 BT_S1     cluster_4             0.526  55611 0.164    334 0.000695
##   5 BT_S2     cluster_1             0.847  72771 0.0581   382 0.000215
##   6 BT_S2     cluster_2             0.848  17391 0.0558   187 0.000423
##   7 BT_S2     cluster_3             0.828 128271 0.0678   507 0.000189
##   8 BT_S4     cluster_1             0.583  67896 0.132    369 0.000506
##   9 BT_S4     cluster_2             0.792 172578 0.0796   588 0.000192
##  10 BT_S4     cluster_3             0.4       820 0.187     41 0.00653
##  11 BT_S4     cluster_4             0.688  50721 0.120    319 0.000534
##  12 BT_S6     cluster_1             0.545    136 0.172     17 0.0147
##  13 BT_S6     cluster_2             0.667   9045 0.110    135 0.00116
##  14 BT_S6     cluster_3             0.854   6105 0.0716   111 0.000917
##  15 BT_S6     cluster_4             0.778   2850 0.111     76 0.00208
```

```
cluster_names <-
    c("Immune\ncells", "Oligo-\ndendrocytes", "Mixed\n(neoplastic)",
      "Neoplastic",
      "Immune\ncells", "Neoplastic", "Mixed\n(neoplastic)",
      "Immune\ncells 1", "Immune\ncells 2", "Immune\ncells 3", "Mixed",
      "Neoplastic", "Mixed\n(neoplastic)", "Immune\ncells 1",
      "Immune\ncells 2")
gbm_patients_and_clusters$cluster <- cluster_names
bar_sem(gbm_patients_and_clusters, "cluster",
        "../results/figures/heterogeneity.gbm.patients_and_clusters.png",
        facets = TRUE, width = 11)

# ANOVA and Tukey's HSD
for (patient in c("BT_S1", "BT_S2", "BT_S4", "BT_S6")) {
    current <- gbm_filtered %>%
        filter_("patient_1" == patient) %>%
        filter_("cluster_1" == "cluster_2")
    current <- gbm_filtered[gbm_filtered$patient_1 == patient, ]
    current$cluster_1 <- factor(current$cluster_1)
    anova_gbm <- aov(lm(1 - distance ~ cluster_1, data = current))
    print(patient)
    print(summary(anova_gbm))
    tukey_gbm <- TukeyHSD(anova_gbm)
    print(tukey_gbm)
}
```

```
## [1] "BT_S1"
##                 Df Sum Sq Mean Sq F value Pr(>F)
## cluster_1        3     30   10.03   328.9 <2e-16 ***
## Residuals   108807   3317    0.03
## ---
## Signif. codes:  0 '***' 0.001 '**' 0.01 '*' 0.05 '.' 0.1 ' ' 1
##   Tukey multiple comparisons of means
##     95% family-wise confidence level
##
## Fit: aov(formula = lm(1 - distance ~ cluster_1, data = current))
##
## $cluster_1
##                              diff          lwr          upr     p adj
```

```
## cluster_2-cluster_1 -0.044125469 -0.049649785 -0.038601153 0.0000000
## cluster_3-cluster_1 -0.047862262 -0.054079492 -0.041645031 0.0000000
## cluster_4-cluster_1 -0.001867295 -0.005725694  0.001991104 0.5992038
## cluster_3-cluster_2 -0.003736792 -0.010434360  0.002960775 0.4784143
## cluster_4-cluster_2  0.042258174  0.037665713  0.046850635 0.0000000
## cluster_4-cluster_3  0.045994966  0.040588804  0.051401128 0.0000000
##
## [1] "BT_S2"
##                 Df Sum Sq Mean Sq F value Pr(>F)
## cluster_1        2      5  2.5045   559.9 <2e-16 ***
## Residuals   578347   2587  0.0045
## ---
## Signif. codes:  0 '***' 0.001 '**' 0.01 '*' 0.05 '.' 0.1 ' ' 1
##   Tukey multiple comparisons of means
##     95% family-wise confidence level
##
## Fit: aov(formula = lm(1 - distance ~ cluster_1, data = current))
##
## $cluster_1
##                             diff          lwr          upr p adj
## cluster_2-cluster_1 -0.003547724 -0.004160707 -0.002934741     0
## cluster_3-cluster_1 -0.006876347 -0.007362518 -0.006390175     0
## cluster_3-cluster_2 -0.003328623 -0.003874634 -0.002782611     0
##
## [1] "BT_S4"
##                 Df Sum Sq Mean Sq F value Pr(>F)
## cluster_1        3   6613    2204  124959 <2e-16 ***
## Residuals   866582  15288       0
## ---
## Signif. codes:  0 '***' 0.001 '**' 0.01 '*' 0.05 '.' 0.1 ' ' 1
##   Tukey multiple comparisons of means
##     95% family-wise confidence level
##
## Fit: aov(formula = lm(1 - distance ~ cluster_1, data = current))
##
## $cluster_1
##                            diff         lwr         upr p adj
## cluster_2-cluster_1  0.18455835  0.18370901  0.18540769     0
## cluster_3-cluster_1 -0.08581622 -0.08781776 -0.08381469     0
## cluster_4-cluster_1  0.11727448  0.11629415  0.11825481     0
## cluster_3-cluster_2 -0.27037457 -0.27239291 -0.26835623     0
## cluster_4-cluster_2 -0.06728387 -0.06829806 -0.06626967     0
## cluster_4-cluster_3  0.20309070  0.20101384  0.20516756     0
##
## [1] "BT_S6"
##                Df Sum Sq Mean Sq F value Pr(>F)
## cluster_1       3   84.7   28.22    1398 <2e-16 ***
## Residuals   57287 1156.1    0.02
## ---
## Signif. codes:  0 '***' 0.001 '**' 0.01 '*' 0.05 '.' 0.1 ' ' 1
##   Tukey multiple comparisons of means
##     95% family-wise confidence level
##
## Fit: aov(formula = lm(1 - distance ~ cluster_1, data = current))
```

```
##
## $cluster_1
##                              diff         lwr         upr p adj
## cluster_2-cluster_1  0.09885407  0.09001577  0.10769238     0
## cluster_3-cluster_1  0.16910733  0.16026819  0.17794647     0
## cluster_4-cluster_1  0.13976838  0.13074768  0.14878907     0
## cluster_3-cluster_2  0.07025326  0.06667587  0.07383064     0
## cluster_4-cluster_2  0.04091430  0.03690928  0.04491933     0
## cluster_4-cluster_3 -0.02933895 -0.03334582 -0.02533208     0
```

GBM: cell types per cluster

```
# Initialise dataframe for holding patient and cluster data
all_patients_and_clusters <- data.frame()

# Get cell type metadata
cell_type_metadata <- metadata_gbm[c("SRR", "cell_type")]

# Read and add data
for (patient in c("BT_S1", "BT_S2", "BT_S4", "BT_S6")) {

    # Read data and merge with metadata
    file <- paste0("../results/clustering/clusters/gbm/clusters.", patient,
                   ".txt")
    data <- read.table(file, header = TRUE, sep = "\t",
                       stringsAsFactors = FALSE)
    data <- merge(data, cell_type_metadata, by.x = "id", by.y = "SRR")

    # Add patient column
    data$patient <- gsub("BT_", "", patient)
    names(data)[1] <- "SRR"

    # Collate
    all_patients_and_clusters <- rbind(all_patients_and_clusters, data)
}

# Summarise
all_patients_and_clusters$cell_type <-
    gsub("_", " ", all_patients_and_clusters$cell_type)
all_patients_and_clusters_summary <- all_patients_and_clusters %>%
    group_by(patient, cluster, cell_type) %>%
    summarise(count = n()) %>%
    mutate(prop = count / sum(count) * 100)

# Plot
gg <- ggplot(all_patients_and_clusters_summary,
             aes(x = cluster, y = prop, fill = cell_type)) +
    geom_bar(stat   = "identity",
             colour = "white") +
    theme_bw() +
    facet_wrap(~ patient, nrow = 1) +
    labs(x    = "Cluster",
         y    = "Proportion of cell type in cluster (%)",
         fill = NULL) +
    theme(panel.grid.major = element_blank(),
          panel.grid.minor = element_blank(),
          strip.background = element_blank(),
          panel.border     = element_rect(colour = "black")) +
    scale_fill_manual(values = c("#19a45e", "#4e8ce4", "#999999", "#0d5933",
                                 "#4ee499", "#a6f2cc", "#19a4a4"))

# Save to file
ggsave("../results/figures/gbm_cluster_cell_types.png", dpi = 300,
```

```
       height = 5, width = 15)
```

GBM: pseudo-profiles

```r
# Function for reading pseudo-profiles
pseudo_patient <- function(patient) {

    # Initialise patient-specific pseudo-profile and get relevant files
    pseudo <- data.frame(stringsAsFactors = FALSE)
    current <- paste0("pseudo_", patient, "_")
    pseudo_dir <- "../results/pseudo_profiles/gbm/"
    files <- list.files(pseudo_dir, pattern = current)

    for (file in files) {

        # Read profile
        profile <- read.table(paste0(pseudo_dir, file), header = TRUE,
                              stringsAsFactors = FALSE, sep ='\t', quote = "")

        # Add cluster and patient name
        cluster_name <- gsub(current, "", gsub(".profile.txt", "", file))
        profile$cluster <- paste("Cluster", cluster_name)
        profile$patient <- patient

        # Add to patient-specific profile
        pseudo <- rbind(pseudo, profile)
    }

    # Return final pseudo-profile
    return(pseudo)
}

# Read and collate pseudo-profiles
ps1 <- pseudo_patient("BT_S1")
ps2 <- pseudo_patient("BT_S2")
ps4 <- pseudo_patient("BT_S4")
ps6 <- pseudo_patient("BT_S6")
pseudo <- do.call("rbind", list(ps1, ps2, ps4, ps6))

# Calculate matches/mismatches in pseudo-profiles
pseudo_summary <- pseudo %>%
    filter(count != 1) %>%
    distinct(patient, cluster, chr, pos, genotype, ENSGID, impact) %>%
    group_by(patient, cluster, chr, pos, ENSGID, impact) %>%
    summarise(count = n()) %>%
    arrange(desc(count)) %>%
    mutate(match = ifelse(count == 1, "match", "mismatch"))

clusters <- pseudo_summary %>%
    group_by(patient, cluster, impact, match) %>%
    summarise(count = n()) %>%
    mutate(prop = count/sum(count))

# Prepare for plotting
clusters$impact = factor(clusters$impact,
```

```r
                         levels=c("HIGH", "MODERATE", "LOW", "MODIFIER"))
clusters[clusters$match == "match", "match"] = "Match"
clusters[clusters$match == "mismatch", "match"] = "Mismatch"

# Plot
gg = ggplot(clusters, aes(x = impact, y = prop, fill = match)) +
    geom_bar(stat = "identity") +
    geom_text(aes(label = count),
              colour   = "white",
              position = position_stack(vjust = 0.5)) +
    facet_grid(patient ~ cluster) +
    theme_bw() +
    scale_fill_manual(values=c("#0d2d59", "#1954a6")) +
    labs(x = NULL, y = "Proportion in category (%)", fill = NULL) +
    theme(panel.grid.major = element_blank(),
          panel.grid.minor = element_blank(),
          strip.background = element_blank(),
          panel.border     = element_rect(colour = "black"))
ggsave("../results/figures/pseudo_impacts.png", gg, dpi = 300, height = 12,
       width = 12)
```

GBM: enrichment analysis

```
# Get biomaRt info
mart <- useMart("ensembl", dataset = "hsapients_gene_ensemble")
attributes <- c("ensembl_transcript_id",
                "ensembl_gene_id",
                "entrezgene",
                "hgnc_symbol",
                "gene_biotype")
info <- getBM(attributes = attributes, mart = mart)

# Remove unnecessary columns, duplicates and missing values
info <- unique(info[c("ensembl_gene_id", "entrezgene")])
info <- info[!is.na(info$entrezgene), ]

# Convert to character
info$entrezgene <- as.character(info$entrezgene)
```

```
# Loop over patients
for (cur_patient in c("BT_S1", "BT_S2", "BT_S4", "BT_S6")) {

    # Get current patient data
    current_patient <- pseudo_summary %>%
        filter(patient == cur_patient)

    # Get available patient clusters
    current_clusters <- unique(current_patient$cluster)

    # Loop over patient clusters
    for (cur_cluster in current_clusters) {

        # Current cluster
        current <- current_patient %>%
            filter(cluster == cur_cluster & match == "match") %>%
            distinct(ENSGID)

        # Write to file
        output <- paste0("../results/pseudo_profiles/gbm/genes/genes.",
                         cur_patient, "_", gsub("luster ", "", cur_cluster),
                         ".txt")
        write.table(current, output, sep = "\t", row.name = FALSE,
                    quote = FALSE)
    }
}
```

```
# List gene files
in_dir <- "../results/pseudo_profiles/gbm/genes"
files <- list.files(in_dir, pattern = paste0("genes.*", ".txt"))

# Loop over each file
for (file in files) {

    # Read gene data and merge with biomaRt info
    data <- read.table(file, header = TRUE, sep = "\t", quote = "",
```

```
                        stringsAsFactors = FALSE)
    data <- merge(data, info, by.x = "ENSGID", by.y = "ensembl_gene_id")
    data <- data[!duplicated(data$entrezgene), ]

    # Perform GO enrichment
    enrich <- enrichGO(gene          = data$entrezgene,
                       universe      = info$entrezgene,
                       OrgDb         = org.Hs.eg.db,
                       keyType       = "ENTREZID",
                       ont           = "BP",
                       pAdjustMethod = "BH",
                       pvalueCutoff  = 0.01,
                       readable      = TRUE)

    # Simplify the GO-terms and select the top 10 terms
    enrich <- simplify(enrich,
                       cutoff     = 0.7,
                       by         = "p.adjust",
                       select_fun = min)
    enrich <- head(enrich, 10)

    # Save to file
    write.table(enrich,
                gsub("genes.", "enrich.", file),
                sep       = "\t",
                quote     = FALSE,
                row.names = FALSE)
}
```

```
# Loop over each patient
for (patient in c("BT_S1", "BT_S2", "BT_S4", "BT_S6")) {

    # List all patient enrichment files
    in_dir <- "../results/pseudo_profiles/gbm/genes"
    enrich_files <- list.files(in_dir,
                               pattern = paste0("enrich.", patient, ".*.txt"))

    # Initiate plot list
    plot_list <- list()

    # Loop over files
    for (file in enrich_files) {

        # Read enrichment data
        enrich <- read.table(paste0(in_dir, "/", file), sep = "\t",
                             header = TRUE,# quote = "",
                             stringsAsFactors = FALSE)

        # Get top 10 terms
        enrich <- enrich[order(enrich$p.adjust), ]
        data <- head(enrich, 10)

        # Separate long terms with newlines
        data$Description <- gsub("([^ ]+ [^ ]+) ", "\\1\n", data$Description)
```

```r
        # Set factors for plotting order
        data$Description <- factor(data$Description,
                                   levels = rev(data$Description))

        # Set output and title
        title <- strsplit(file, "\\.")[[1]][2]
        title <- gsub("_C", " Cluster ", title)

        # Plot and add to list
        gg <- ggplot(data, aes(x    = Description,
                               y    = -log10(p.adjust),
                               fill = -log10(p.adjust))) +
            geom_bar(stat = "identity") +
            coord_flip() +
            theme_classic() +
            labs(title = title,
                 x     = NULL,
                 y     = expression(-log[10](FDR))) +
            theme(plot.title = element_text(hjust = 0.5),
                  axis.text  = element_text(size = 7)) +
            scale_fill_gradientn(colours = c("#a6c6f2", "#a6c6f2",
                                             "#0d2d59", "#0d2d59"),
                                 limits  = c(0, 100),
                                 values  = rescale(c(0, 5, 30, 100)),
                                 guide   = FALSE)
        plot_list[[length(plot_list) + 1]] <- gg
    }

    # Plot current patient clusters
    gg <- cowplot::plot_grid(plotlist = plot_list, nrow = 2)
    output <- paste0("../results/figures/enrichment.", patient, ".png")
    ggsave(output, gg, dpi = 300, height = 10, width = 10)
}
```

GBM: driver genes and prognostic markers

```
# Driver mutations
drivers <- read.table("../data/intogen-GBM-drivers-data.tsv", sep = '\t',
                      head = TRUE, stringsAsFactors = FALSE)
drivers <- drivers[drivers$KNOWN_DRIVER == "True", ]
pseudo_summary$patient <- gsub("BT_", "", pseudo_summary$patient)
pseudo_drivers = merge(pseudo_summary, drivers, by.x = "ENSGID", by.y = "GENE")
pseudo_drivers <- pseudo_drivers %>%
    group_by(patient, cluster, match) %>%
    summarise(count = n()) %>%
    mutate(prop = (1 - count/sum(count)) * 100) %>%
    filter(match == "match")
pseudo_drivers$name <- cluster_names
pseudo_drivers$count <- round(pseudo_drivers$prop * pseudo_drivers$count /
    (100 - pseudo_drivers$prop), 0)

gg_drivers <- ggplot(pseudo_drivers, aes(x = name, y = prop)) +
    geom_bar(stat     = "identity",
             fill     = "#1954a6",
             position = position_dodge()) +
    geom_text(aes(label = paste(round(prop, 1), "%")),
              position = position_dodge(0.9),
              vjust    = -0.3) +
    geom_text(data = pseudo_drivers[pseudo_drivers$prop != 0, ],
              aes(label = paste0("(", count, ")")),
              colour   = "white",
              position = position_dodge(0.9),
              vjust    = 1.6) +
    facet_grid(. ~ patient, scales = "free_x") +
    theme_bw() +
    ylim(0, 15) +
    theme(axis.title       = element_text(size = 9),
          panel.grid.major = element_blank(),
          panel.grid.minor = element_blank(),
          strip.background = element_blank(),
          panel.border     = element_rect(colour = "black")) +
    labs(x = NULL,
         y = "Proportion of mismatching variants in driver genes (%)")

# Read HPA prognostic marker data and subset for glioma genes
prognostic <- read.table("../data/pathology.tsv", header = TRUE, sep = "\t",
                         stringsAsFactors = FALSE)
prognostic_gbm <- prognostic[prognostic$Cancer == "glioma", ]
names(prognostic_gbm) <- gsub("\\.\\.\\.", "_", names(prognostic_gbm))
prognostic_gbm <-
    prognostic_gbm[(!is.na(prognostic_gbm$prognostic_favourable) &
                    prognostic_gbm$prognostic_favourable <= 0.05) |
                   (!is.na(prognostic_gbm$prognostic_unfavourable) &
                    prognostic_gbm$prognostic_unfavourable <= 0.05), ]
prognostic_gbm$prognostic <- "favourable"
prognostic_gbm[!is.na(prognostic_gbm$prognostic_unfavourable),
               "prognostic"] <- "unfavourable"
```

```r
# Merge with pseudo profiles and plot
pseudo_prognostic <- unique(merge(pseudo_summary, prognostic_gbm,
                                  by.x = "ENSGID", by.y = "Gene"))
pseudo_prognostic <- pseudo_prognostic %>%
    group_by(patient, cluster, match) %>%
    summarise(count = n()) %>%
    mutate(prop = (1 - count/sum(count)) * 100) %>%
    filter(match == "match")
pseudo_prognostic$name <- cluster_names
pseudo_prognostic$count <- round(pseudo_prognostic$prop *
    pseudo_prognostic$count / (100 - pseudo_prognostic$prop), 0)

# Plot
gg_prognostic <- ggplot(pseudo_prognostic, aes(x = name, y = prop)) +
    geom_bar(stat     = "identity",
             fill     = "#1954a6",
             position = position_dodge()) +
    geom_text(aes(label = paste(round(prop, 1), "%")),
              position = position_dodge(0.9),
              vjust    = -0.3) +
    geom_text(aes(label = paste0("(", count, ")")),
              colour   = "white",
              position = position_dodge(0.9),
              vjust    = 1.6) +
    facet_grid(. ~ patient, scales = "free_x") +
    ylim(0, 15) +
    theme_bw() +
    theme(axis.title       = element_text(size = 9),
          panel.grid.major = element_blank(),
          panel.grid.minor = element_blank(),
          strip.background = element_blank(),
          panel.border     = element_rect(colour = "black")) +
    labs(x = "Cluster",
         y = "Proportion of mismatching variants in prognostic genes (%)")

# Save as figure
gg <- cowplot::plot_grid(gg_drivers, gg_prognostic, nrow = 2,
                         labels = c("A", "B"), vjust = c(1.5, 0.75))
ggsave("../results/figures/gbm_driver_and_prognostic_genes.png", dpi = 300,
       width = 14, height = 8)
```

GBM: common driver genes in neoplastic clusters

```
# Get all missense variants
pseudo_miss <- pseudo %>%
    filter(count != 1) %>%
    filter(effect == "missense_variant") %>%
    distinct(patient, cluster, chr, pos, genotype, ENSGID, impact) %>%
    group_by(patient, cluster, chr, pos, ENSGID, impact) %>%
    summarise(count = n()) %>%
    arrange(desc(count)) %>%
    mutate(match = ifelse(count == 1, "match", "mismatch"))

# Get variants in all neoplastic clusters
pseudo_neoplastic <- pseudo_miss %>%
    distinct(patient, cluster, ENSGID, match) %>%
    filter((patient == "BT_S1" & cluster == "Cluster 4") |
           (patient == "BT_S2" & cluster == "Cluster 2") |
           (patient == "BT_S6" & cluster == "Cluster 1"))
pseudo_neoplastic <- pseudo_neoplastic %>%
    dplyr::select(chr, pos, ENSGID, patient, cluster, match)

# Separate and find common variants across neoplastic clusters
neoplastic_1 <- pseudo_neoplastic %>%
    filter(patient == "BT_S1")
neoplastic_2 <- pseudo_neoplastic %>%
    filter(patient == "BT_S2")
neoplastic_6 <- pseudo_neoplastic %>%
    filter(patient == "BT_S6")
neoplastic <- merge(neoplastic_1, neoplastic_2, by = c("chr", "pos"),
                    suffixes = c("_1", "_2"))
neoplastic <- merge(neoplastic, neoplastic_6, by = c("chr", "pos"))
neoplastic <- neoplastic[!duplicated(neoplastic[c("chr", "pos")]), ]
neoplastic <- neoplastic[c("chr", "pos", "ENSGID")]

# Get top genotype for each patient
neoplastic <- unique(merge(neoplastic,
                           ps1[!duplicated(ps1[c("chr", "pos", "ENSGID")]), ],
                           by = c("chr", "pos", "ENSGID")))
neoplastic <- unique(merge(neoplastic,
                           ps2[!duplicated(ps2[c("chr", "pos", "ENSGID")]), ],
                           by = c("chr", "pos", "ENSGID")))
neoplastic <- unique(merge(neoplastic,
                           ps6[!duplicated(ps6[c("chr", "pos", "ENSGID")]), ],
                           by = c("chr", "pos", "ENSGID")))
neoplastic <- neoplastic[c("chr", "pos", "rsID", "gene", "ENSGID", "impact",
                           "effect", "feature", "biotype", "genotype.x",
                           "genotype.y", "genotype")]
names(neoplastic)[5] <- "ENSGID"
names(neoplastic)[10:12] <- c("genotype_1", "genotype_2", "genotype_6")

# Compare patients
neoplastic$match <- "match"
neoplastic[(neoplastic$genotype_1 != neoplastic$genotype_2) |
```

```
           (neoplastic$genotype_1 != neoplastic$genotype_6) |
           (neoplastic$genotype_2 != neoplastic$genotype_6), "match"] <-
               "mismatch"

# Add known/unknown
neoplastic$known <- "known"
neoplastic[neoplastic$rsID == "None", "known"] <- "unknown"

# Summarise
neoplastic %>%
    group_by(known, match) %>%
    summarise(count = n()) %>%
    mutate(prop = round(count / sum(count) * 100, 1))
```

```
## # A tibble: 4 x 4
## # Groups:   known [2]
##   known   match    count  prop
##   <chr>   <chr>    <int> <dbl>
## 1 known   match      142  87.1
## 2 known   mismatch    21  12.9
## 3 unknown match        2  66.7
## 4 unknown mismatch     1  33.3
```

```
neoplastic %>%
    filter(known == "unknown")
```

```
##     chr      pos rsID    gene          ENSGID   impact           effect
## 1 chr12 46363770 None SLC38A2 ENSG00000134294 MODERATE missense_variant
## 2 chr17 82938057 None    TBCD ENSG00000141556 MODERATE missense_variant
## 3  chr6 29725552 None   HLA-F ENSG00000204642 MODERATE missense_variant
##      feature        biotype genotype_1 genotype_2 genotype_6    match
## 1 transcript protein_coding         GG         GG         GG    match
## 2 transcript protein_coding         GG         GG         GG    match
## 3 transcript protein_coding         GG         GG         AG mismatch
##     known
## 1 unknown
## 2 unknown
## 3 unknown
```

```
# Save all to file
output <- "../results/pseudo_profiles/gbm/neoplastic_variants.txt"
write.table(neoplastic, output, sep = "\t", row.name = FALSE,
            quote = FALSE)
```

Session info

```
## R version 3.5.1 (2018-07-02)
## Platform: x86_64-apple-darwin13.4.0 (64-bit)
## Running under: macOS  10.14.3
##
## Matrix products: default
## BLAS/LAPACK: /Users/erik.fasterius/projects/scRNA-seq/conda-env/lib/R/lib/libRblas.dylib
##
```

```
## locale:
## [1] en_GB.UTF-8/en_GB.UTF-8/en_GB.UTF-8/C/en_GB.UTF-8/en_GB.UTF-8
##
## attached base packages:
## [1] parallel  stats4    stats     graphics  grDevices utils     datasets
## [8] methods   base
##
## other attached packages:
##  [1] clusterProfiler_3.10.1      biomaRt_2.38.0
##  [3] reshape2_1.4.3              seqCAT_1.4.1
##  [5] VariantAnnotation_1.28.3    Rsamtools_1.34.0
##  [7] Biostrings_2.50.2           XVector_0.22.0
##  [9] SummarizedExperiment_1.12.0 DelayedArray_0.8.0
## [11] BiocParallel_1.16.6         matrixStats_0.54.0
## [13] Biobase_2.42.0              GenomicRanges_1.34.0
## [15] GenomeInfoDb_1.18.1         IRanges_2.16.0
## [17] S4Vectors_0.20.1            BiocGenerics_0.28.0
## [19] tidyr_0.8.3                 dplyr_0.8.0.1
## [21] scales_1.0.0                ggplot2_3.1.0
##
## loaded via a namespace (and not attached):
##  [1] bitops_1.0-6             enrichplot_1.2.0
##  [3] bit64_0.9-7              RColorBrewer_1.1-2
##  [5] progress_1.2.0           httr_1.4.0
##  [7] UpSetR_1.3.3             tools_3.5.1
##  [9] utf8_1.1.4               R6_2.4.0
## [11] DBI_1.0.0                lazyeval_0.2.2
## [13] colorspace_1.4-1         withr_2.1.2
## [15] tidyselect_0.2.5         gridExtra_2.3
## [17] prettyunits_1.0.2        bit_1.1-14
## [19] compiler_3.5.1           cli_1.1.0
## [21] xml2_1.2.0               labeling_0.3
## [23] triebeard_0.3.0          rtracklayer_1.42.1
## [25] ggridges_0.5.1           stringr_1.4.0
## [27] digest_0.6.18            rmarkdown_1.11
## [29] DOSE_3.8.2               pkgconfig_2.0.2
## [31] htmltools_0.3.6          BSgenome_1.50.0
## [33] rlang_0.3.1              RSQLite_2.1.1
## [35] gridGraphics_0.3-0       farver_1.1.0
## [37] jsonlite_1.6             GOSemSim_2.8.0
## [39] RCurl_1.95-4.12          magrittr_1.5
## [41] ggplotify_0.0.3          GO.db_3.7.0
## [43] GenomeInfoDbData_1.2.0   Matrix_1.2-16
## [45] fansi_0.4.0              Rcpp_1.0.1
## [47] munsell_0.5.0            viridis_0.5.1
## [49] stringi_1.4.3            yaml_2.2.0
## [51] ggraph_1.0.2             MASS_7.3-51.1
## [53] zlibbioc_1.28.0          plyr_1.8.4
## [55] qvalue_2.14.1            grid_3.5.1
## [57] blob_1.1.1               ggrepel_0.8.0
## [59] DO.db_2.9                crayon_1.3.4
## [61] lattice_0.20-38          cowplot_0.9.4
## [63] splines_3.5.1            GenomicFeatures_1.34.1
## [65] hms_0.4.2                knitr_1.22
```

```
## [67] pillar_1.3.1             fgsea_1.8.0
## [69] igraph_1.2.4             fastmatch_1.1-0
## [71] XML_3.98-1.19            glue_1.3.1
## [73] evaluate_0.13            data.table_1.12.0
## [75] urltools_1.7.2           tweenr_1.0.1
## [77] gtable_0.2.0             purrr_0.3.2
## [79] polyclip_1.10-0          assertthat_0.2.0
## [81] xfun_0.5                 ggforce_0.2.1
## [83] europepmc_0.3            viridisLite_0.3.0
## [85] tibble_2.1.1             rvcheck_0.1.3
## [87] GenomicAlignments_1.18.1 AnnotationDbi_1.44.0
## [89] memoise_1.1.0
```
